# Association Between Air Pollution and Monday Peak Mortality From Acute Myocardial Infarction

**DOI:** 10.1016/j.jacadv.2025.102378

**Published:** 2025-12-17

**Authors:** Ruben Lévy, Laurent Lévy, Joan Ballester, Zhao-Yue Chen, François R. Herrmann, Antonio Gasparrini, Hicham Achebak

**Affiliations:** a Department of Rehabilitation and Geriatrics, Medical School of the University of Geneva, Geneva, Switzerland; b Division of Geriatrics, Department of Rehabilitation and Geriatrics, Geneva University Hospitals, Thônex, Switzerland; c France Cohortes, ISGlobal, Barcelona, Spain; d Environment & Health Modelling (EHM) Lab, Department of Public Health Environments and Society, London School of Hygiene & Tropical Medicine (LSHTM), London, United Kingdom; e Inserm, France Cohortes, Paris, France

**Keywords:** acute myocardial infarction, air pollution, Monday peak, mortality, Spain

## Abstract

**Background:**

Mortality from acute myocardial infarction (AMI) exhibits a weekly pattern, with a peak occurring on Mondays. Although several hypotheses have been proposed, the effect of air pollution on this peak has not yet been investigated.

**Objectives:**

The purpose of this study was to investigate if daily variation in major air pollutants modifies the risk of AMI mortality on Monday.

**Methods:**

A time-series study analyzed 260,320 AMI deaths in Spain from 2004 to 2018, using records from the Spanish National Institute of Statistics. Air pollution (ie, PM_2.5_, PM_10_, nitrogen dioxide, and ozone) levels were estimated across the mainland Spain and the Balearic Islands. Regression models and multilevel meta-analysis were used to assess the weekly variation in AMI mortality and the association between air pollution and the Monday excess in AMI mortality.

**Results:**

AMI mortality varied throughout the week, peaking on Monday (+3.7% above the weekly average). Particulate air pollution (PM_2.5_, PM_10_) also showed a weekly pattern, with lower levels on weekends than weekdays. A significant increase in PM_2.5_ and PM_10_ levels was observed from Sunday to Monday (12.6% and 12.0%, respectively), but not between other weekdays. After adjustment for inter-weekly baseline mortality changes, the intra-weekly pattern in AMI mortality was not modified by either the absolute level or the inter-day change in air pollutants. In addition, no association was found between air pollution and the Monday peak in AMI mortality.

**Conclusions:**

Our results suggest that the excess AMI mortality observed on Mondays in the Spanish population is not explained by concurrent variations in major air pollutants.

Acute myocardial infarction (AMI) is the leading cause of cardiovascular death worldwide.^1^ Epidemiological studies have shown that the incidence of both AMI and AMI mortality varies according to the day of the week, with a peak on Monday and a minimum on Sunday.^2^, ^3^, ^4^, ^5^ This temporal or circaseptan distribution—that is, the rhythmic occurrence of a peak every 7 days—is commonly attributed to cyclical cardiovascular triggers related to social rhythms (eg, the stress of returning to work on Mondays,^2^ the lag effect of weekend excessive alcohol consumption^6^) or internal biological rhythms (eg, circadian rhythm disalignment,^7^ or a hypothetical weekly biological clock).^8^ However, many uncertainties remain about its underlying causes, as association studies are lacking and other environmental risk factors also prone to periodicity, such as air pollution, have long been overlooked.

In fact, a number of findings in the literature point to air pollution as a plausible candidate that could account for the Monday peak. Firstly, traffic and industrial activities are a major source of coarse (PM_10_) and fine (PM_2.5_) particles,^9^ which are today considered among the largest cardiovascular risk factors in population studies.^10^ Secondly, environmental analyses have highlighted weekly variations in air pollutant levels, with weekends being less polluted than the start of the week.^11^ Lastly, exposure to acute variation in particulate air pollution significantly increases the risk of AMI within a very short time frame (1-6 hours).^12^^,^^13^

In this study, we therefore tested the hypothesis that daily variation in major air pollutants (PM_10_, PM_2.5_, nitrogen dioxide [NO_2_], ozone [O_3_]) modifies the risk of AMI mortality on Mondays. To assess this association, we examined daily mortality and air pollution data from Spain over a 15-year period (2004-2018).

## Methods

### Data sources

This country-wide time-series study used data on deaths and air pollution (ie, PM_2.5_, PM_10_, NO_2_, and O_3_) from 48 provinces in mainland Spain and the Balearic Islands (Supplemental Figure 1), representing an average population of about 43.6 million people (95.2% of the total Spanish population).

Individual death records were provided by the Spanish National Institute of Statistics upon request. The database included, among others, the following variables for the deceased: date of death, sex, age, province of residence, and the cause of death coded according to the International Classification of Diseases-10th Revision (ICD-10). Individual death records were aggregated by date of decease and province of residence in order to perform the statistical analysis described below.

As previously described,^14^ daily mean concentrations of PM_2.5_, PM_10_, NO_2_, and daily maximum 8-hour averages of O_3_ across Spain were estimated using a Quantile Machine Learning model framework at a spatial resolution of 10 km × 10 km. The model development involved the integration of various data sources, including ground monitoring measurements, fine and course mode Aerosol Optical Depth, climate and air quality reanalysis data, and geographical features (eg, land use, topography, road traffic). The model was trained using data from across Europe, covering the period from January 1, 2003, to December 31, 2020. To assess the accuracy of the model, we conducted a 10-fold validation. The results showed good performance, with correlation coefficients of 0.80, 0.79, 0.79, and 0.90 for PM_2.5_, PM_10_, NO_2,_ and O_3_, respectively, when compared with site observations in Europe. The normalized root mean square error for PM_2.5_, PM_10_, NO_2,_ and O_3_ predictions in Europe were found to be 1.84%, 2.07%, 8.99%, and 3.35%, respectively, compared to site observations. Air pollution data were transformed into provincial estimates by weighting the values with 1 km × 1 km gridded population counts for the year 2011 from Spanish National Institute of Statistics.^15^

### Statistical analysis

The statistical analysis was performed with R software version 4.5.1 (R Foundation for Statistical Computing) and consisted of 2 steps that are described below.

#### First stage time-series regression

We used time-series regression models to assess: 1) the weekly variation in AMI (ICD-10: I21-I22) mortality and the contribution of air pollution; and 2) the association between air pollution and the Monday excess in AMI deaths.

First, the weekly cycle of AMI deaths in each Spanish province was estimated using a daily time-series quasi-Poisson regression model including a categorical variable of day of the week, together with a spline of time with 8 degrees of freedom to account for seasonal and long-term trends:

(1) log(μ)=α+dow+ns(time)

where *μ* denotes the expected number of AMI deaths; α the intercept; *dow* the categorical variable of day of the week; and *ns* the natural cubic B-spline. Next, in order to evaluate the role of air pollution in increasing AMI mortality, we introduced, respectively, in Equation 1, a linear term for: 1) daily mean air pollution concentrations at lags 0 to 2; and 2) inter-day pollution variation (defined as the difference between the daily mean concentration of 2 consecutive days). Moreover, to examine effect modification by season (winter [December-March] and summer [June-September]), we introduced an interaction between *dow* and a binary variable of season.

Separately, the association between air pollution and the Monday excess in AMI mortality across Spanish provinces was examined through a conditional quasi-Poisson regression model, which was fitted on a subset of data including only Sunday and Monday observations. The model included a stratum variable defined by week and year, a categorical variable of day of the week (1 = Sunday [week day of minimum AMI mortality], 2 = Monday [week day of maximum AMI mortality]), and, respectively, a linear term for daily mean air pollution concentration and inter-day pollution variation (ie, difference in air pollution levels between Monday and Sunday):

(2) log(μ)=stratum+dow+pollution

This modeling strategy isolated Monday–Sunday differences in mortality by controlling for inter-week variability through fixed effects.

The models described above provided sets of coefficients representing the province-specific estimates, as well as their (co)variance matrices.

#### Second-stage meta-analysis

In the second stage, we used multivariate (in the analysis of the weekly variation in AMI mortality) and univariate (in the analysis of the association between air pollution and Monday peak in AMI deaths) multilevel meta-analysis^16^ to pool the province-specific estimates. This allowed us to derive the average associations across the whole country, which were reported either as relative risk (RR) of death or as percent increase in the risk of mortality, with 95% empirical CI. The meta-analytical model can be written as:

(3) $$\theta_{\text{ij}}=\beta_{0}+\gamma_{i}Y_{i}+\delta_{j}Z_{\text{ij}}+\varepsilon_{\text{ij}}\text{,}$$

$$\gamma_{i}∼\;N\left(0,\tau_{1}^{2}\right),\delta_{j}∼\;N\left(0,\tau_{2}^{2}\right),\varepsilon_{\text{ij}}∼\;N\left(0,s_{\text{ij}}^{2}\right)$$

where $\theta_{\text{ij}}$ denotes the coefficients representing AMI mortality in province *j* (n = 48) within region *i* (n = 16); $\beta_{0}$ the pooled effect; $Y_{i}$ the matrix of region indicators with random coefficients $\gamma_{i}$; $Z_{\text{ij}}$ the matrix of province indicators with random coefficients $\delta_{j}$; and $\varepsilon_{\text{ij}}$ the error term distributed with province-specific (co)variance matrices $s_{\text{ij}}^{2}$. The random coefficients had unstructured (co)variance matrices $\tau^{2}$. Residual heterogeneity was tested and quantified using the multivariate extension of the Cochran Q test and the *I*^*2*^ statistic, respectively.

Finally, in order to assess whether the associations varied by degree of urbanization or rurality, we categorized the provinces into 4 groups (ie, quartiles) based on population density data (ie, proxy of degree of urbanization/rurality), and then fitted the meta-analytical model described above for each group.

## Results

From 2004 to 2018, there were 260,320 deaths (mean [IQR] age, 77.4 [16.0] years; 41.6% women) from AMI in the Spanish population, accounting for 52.8% and 14.9% of ischemic heart disease (ICD-10: I20-I25) and total cardiovascular disease (ICD-10: I00-I99), respectively. Crude mortality rates for AMI largely decreased over time (from 5.4 deaths per 10,000 in 2004-3.0 deaths in 2018) (Supplemental Figure 1A), despite an aging population. The national average (interprovince range) of population-weighted daily mean concentrations of air pollutants was 10.4 μg/m^3^ (7.1-15.8) for PM_2.5_, 21.3 μg/m^3^ (12.0-32.6) for PM_10_, 12.7 μg/m^3^ (10.0-17.2) for NO_2_, and 79.6 μg/m^3^ (72.6-85.1) for O_3_. Except for O_3_, population exposure to air pollution showed a clear reduction during the study period (Supplemental Figures 1B to 1E).

**Figure 1:**
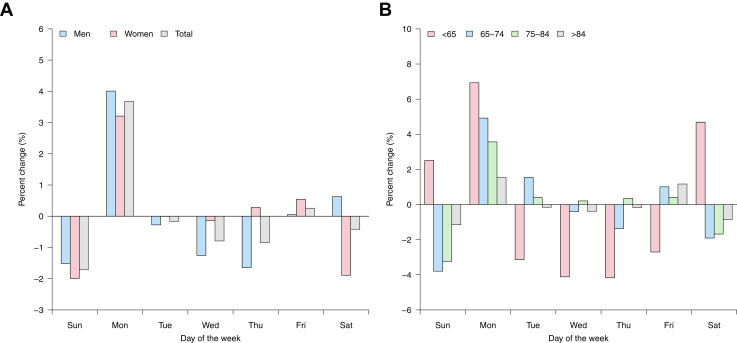
**Weekly Variation of Mortality From AMI** The values represent the percent change with regard to the weekly average (A) by sex, (B) by age. AMI = acute myocardial infarction.

Figure 1 displays the weekly variation in AMI mortality, expressed as the percentage change (%) of mortality on each day of the week with regard to the weekly average. Overall, Sunday exhibited the lowest incidence of AMI mortality (−1.7% below the weekly average), whereas Monday showed the highest incidence (+3.7% above the weekly average), corresponding to an estimated relative increase in AMI mortality of 5.4% from Sunday to Monday. The remaining days of the week were marked by a moderate and stable number of AMI deaths, with deviations from the weekly average ranging between −0.8% and 0.3%. The Monday peak was more pronounced in men (Figure 1A), and its magnitude decreased with age (Figure 1B). In addition, the Sunday trough was more pronounced in women, and its magnitude decreased with age, except for people younger than 65 years. The Monday peak was also less prominent for other ischemic heart diseases and was not found for other (non)cardiovascular diseases (Supplemental Figure 3).

**Figure 2:**
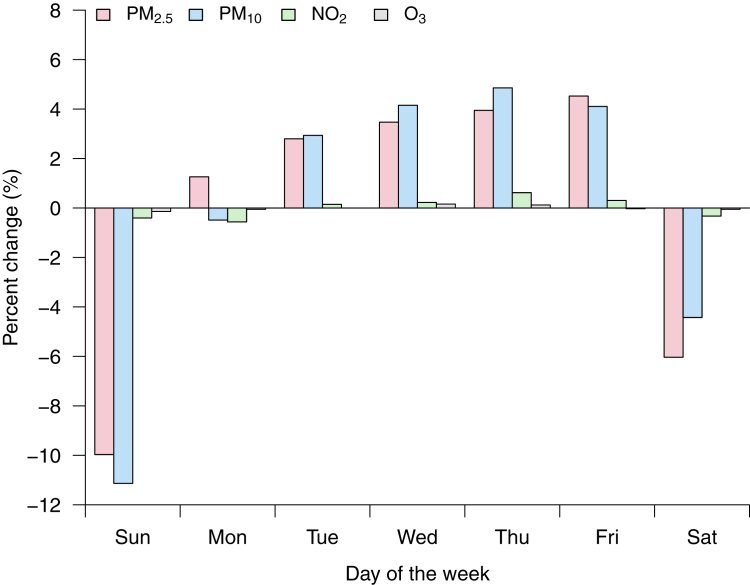
**Weekly Variation of Air Pollution Levels** The values represent the percent change with regard to the weekly average. NO^2^ = nitrogen dioxide; O^3^ = ozone; PM_2.5_ = fine particulate matter (aerodynamic diameter <2.5 μm); PM_10_ = coarse particulate matter (aerodynamic diameter <10 μm).

Figure 2 shows the weekly variation of the air pollutants analyzed in this study, namely PM_2.5_, PM_10_, NO_2_, and O_3_. NO_2_ and O_3_ levels remained stable throughout the week, while particulate air pollution (PM_2.5_, PM_10_) showed greater variability depending on the day of the week. PM_2.5_ and PM_10_ levels were lower on weekends than on weekdays, with a minimum on Sundays (−10% [−1.0 μg/m^3^] and −11% [−2.3 μg/m^3^], respectively, relative to the weekly mean). The relative increase in PM_2.5_ and PM_10_ levels between Sunday and Monday was 12.6% and 12.0%, or in absolute terms 1.2 μg/m^3^ and 2.2 μg/m^3^. Particulate concentrations continued to rise over the remaining working days, albeit with lower inter-day variability.

**Figure 3:**
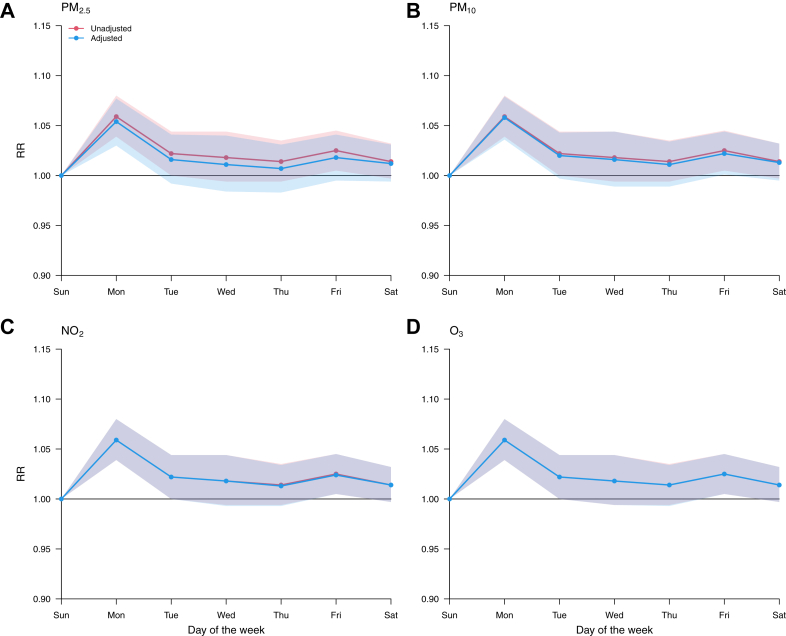
**Weekly Variation of the Risk of AMI Mortality Before and After Adjustment for Daily Air Pollution** RR curves are computed using Sunday as a reference. RR = relative risk; other abbreviations as in Figures 1 to 3.

**Figure 4:**
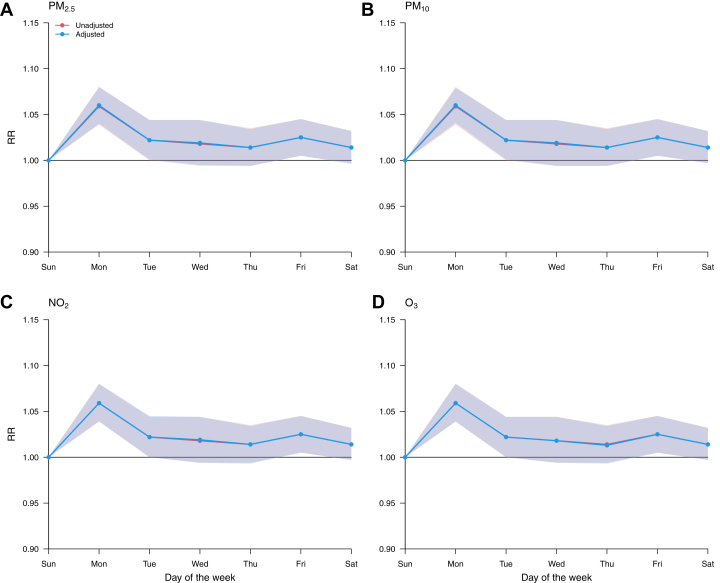
**Weekly Variation of the Risk of AMI Mortality Before and After Adjustment for Inter-Day Change in Air Pollution** RR curves are computed using Sunday as a reference. Abbreviations as in Figure 1, Figure 2, Figure 3.

Figures 3 and 4 depict the RR of AMI mortality on each day of the week compared to the minimum mortality week day derived from the multivariate meta-analysis. Before adjustment for air pollutants, the RR of death was highest on Monday (RR = 1.060 [95% empirical CI: 1.040-1.080]), and progressively declined to reach a minimum on Sunday (ie, the minimum mortality week day). After adjustment for either daily mean air pollution (PM_2.5_, PM_10_, NO_2_, O_3_) at lag 0 (Figure 3) and lags 1 to 2 (Supplemental Figure 4), or inter-day variation in air pollutants (Figure 4), the weekly pattern in AMI mortality hardly changed. Moreover, the weekly cycle in AMI mortality was not modified by season (Supplemental Figures 5 and 6) nor the degree of urbanization/rurality of the province (Supplemental Figure 7 and 8).

The results of the analysis focusing exclusively on the association between air pollution and the Monday excess in AMI mortality are reported in Table 1. We found no statistically significant association between daily level of air pollutants and the Monday peak in AMI mortality: a 1 μg/m3 increase in PM_2.5_, PM_10_, NO_2_, and O_3_ increased the mortality risk on Monday by 0.03% (−1.05-1.11), 0.11% (−0.18-0.39), 0.10% (−0.70-0.91), and 0.25% (−0.09-0.60), respectively. Note that this lack of association was independent of the season (Table 1) and the degree of urbanization/rurality of the province (Supplemental Table 1). Moreover, when testing whether the change in pollution (between Sunday and Monday), rather than its absolute level, is associated with excess mortality on Monday, we yielded identical results (Table 1).

**Table 1:** Monday Change in AMI Mortality Risk per 1-μg-per-Cubic-Meter Increase in Air Pollutants

Pollutant	Annual	Winter Season	Summer Season
PM_2.5_	0.03 (−1.05 to 1.11)	0.25 (−1.27 to 1.79)	0.58 (−1.67 to 2.88)
PM_10_	0.11 (−0.18 to 0.39)	0.11 (−0.19 to 0.41)	−0.14 (−0.92 to 0.64)
NO_2_	0.10 (−0.70 to 0.91)	0.12 (−0.51 to 0.75)	0.21 (−1.42 to 1.86)
O_3_	0.25 (−0.09 to 0.60)	0.10 (−0.41 to 0.61)	0.23 (−0.60 to 1.06)

AMI = acute myocardial infarction; NO_2_ = nitrogen dioxide; O_3_ = ozone; PM_2.5_ = fine particulate matter (aerodynamic diameter <2.5 μm); PM_10_ = coarse particulate matter (aerodynamic diameter <10 μm).

The estimates represent the percentage increase in mortality risk along with 95% CI. Winter = December-March; Summer = June-August.

Finally, the results of heterogeneity from the meta-analyses are reported in Supplemental Tables 1 and 2. On the one hand, the weekly variation in AMI mortality (Supplemental Table 2), the multivariate Cochran Q test for heterogeneity was not significant (*P* > 0.05) neither for the unadjusted models nor pollution-adjusted models describing the weekly cycle in AMI mortality. In addition, the *I*^*2*^ statistic indicated that only 7.8% (unadjusted model) and 7.9% to 11.1% (pollution-adjusted models) of the variation in the province-specific estimates was attributable to heterogeneity between provinces. On the other hand, regarding the association between air pollution and Monday peak in AMI mortality, there was evidence of heterogeneity (*P* < 0.05) for all pollutants, although it was moderate (*I*^*2*^ statistic = 38%-44%) (Supplemental Table 3).

## Discussion

This population-based study spanning 15 years examined daily AMI mortality and air pollution across Spain. We observed a weekly variation in AMI deaths, characterized by a minimum on Sunday and a peak on Monday. Particulate air pollution displayed a comparable weekly pattern. However, after adjusting for air pollution exposure, the Monday peak in AMI mortality was not associated with main air pollutants (PM_2.5_, PM_10_, NO_2_, and O_3_) (Central Illustration). To the best of our knowledge, this is the first study to investigate the relationship between an environmental risk factor and the Monday excess in AMI mortality.

**Central Illustration:**
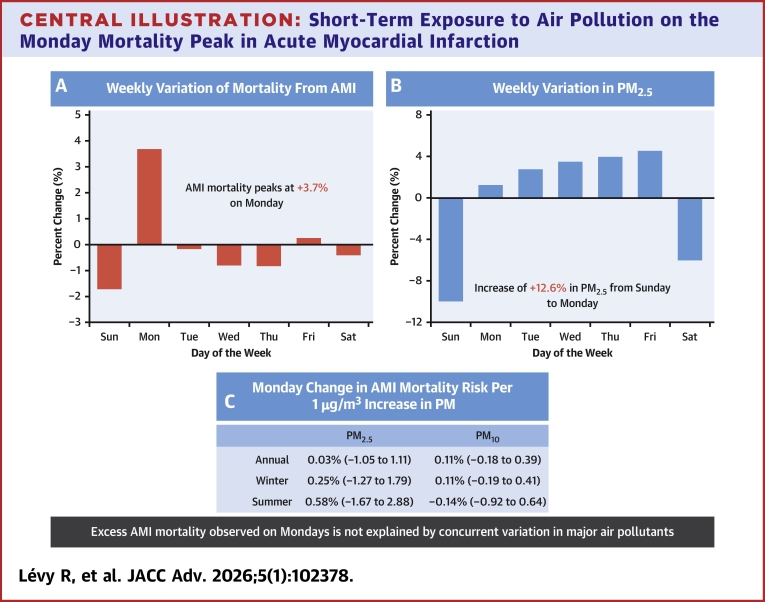
**Short-Term Exposure to Air Pollution on the Monday Mortality Peak in Acute Myocardial Infarction** This time-series analysis of 260,320 AMI deaths in Spain (2004-2018) examined whether air pollution explains Monday mortality peaks. (A) Weekly variation in AMI mortality shows a Monday peak (+3.7% above the weekly average). (B) Weekly variation in particulate matter pollution (PM_2.5_) levels displays a significant increase from Sunday to Monday (+12.6%). (C) The strong variation in PM from Sunday to Monday does not predict the higher risk of AMI mortality on Monday, neither in summer (June-September) nor in winter (December-March). Abbreviations as in Figures 1 and 2.

Since its first description by Rogot et al^17^ in 1976, the Monday peak in AMI mortality and other cardiovascular diseases (eg, ischemic heart disease, ventricular arrhythmias, sudden cardiac death) has been well-documented.^3^^,^^4^ The present study, however, provides insight into several previously debated aspects. First of all, we find a significant excess of AMI mortality of 3.7% on Mondays, which is in the lower range of previously reported estimates.^3^, ^4^, ^5^ Although this mortality excess may appear modest, it is sufficient to result in a negative risk on the days following the peak, probably because of a harvesting effect. Similar findings have been demonstrated in a recent prospective Irish study examining the association between the occurrence of ST-segment elevation myocardial infarction cases and the day of the week.^18^ Furthermore, our subgroup analysis yielded results consistent with those previously observed regarding sex, but not with age. Although the Monday peak is known to affect men more than women,^3^^,^^19^ it is surprising to find that it also occurs in retired people (>65 years). This finding challenges the long-established hypothesis that the first day of the week triggers myocardial infarction primarily in the working population under 65 years of age.^2^ In this group, the Monday peak was preceded by an elevated risk of AMI mortality during the weekend. This pattern, also reported by Evans et al,^20^ could be explained by a number of well-known behavioral cardiovascular risk factors affecting the <65 years age group on weekends, such as physical exertion, excessive alcohol consumption, and recreational drug use.^20^, ^21^, ^22^ Lastly, no Monday increase in mortality from other cardiovascular diseases was noted, contrary to findings reported in several previous population studies.^5^^,^^23^

Despite substantial evidence in the literature suggesting a potential link between air pollution and AMI events, we found no association between air pollution and the Monday peak in AMI mortality. Particulate air pollution (especially PM_2.5_), largely emitted by road traffic, is a major risk factor for AMI in the general population due to its continuous and widespread nature, with an estimated population attributable fraction of 5%.^10^ Increasing evidence indicates that short-term exposure to PM_2.5_ can trigger an AMI within hours,^13^ even at concentrations below the World Health Organization limit values,^24^ probably via sympathetic activation and thrombotic mechanisms.^25^ Considering the rapid fatality of AMI, a mortality peak on Monday, coinciding with a pronounced increase in particulate pollution, would be expected.

## Study limitations

The negative results of our study should be interpreted in the light of several limitations. First, we did not measure hourly air pollution levels, but rather daily averages, due to a lack of data. Indeed, several studies have demonstrated that hourly peaks in air pollution concentration are more strongly associated with major cardiac events than daily averages, which tend to underestimate variations in air pollution throughout the day.^26^ Second, we were not able to control for certain individual characteristics, including cardiovascular risk factors (eg, alcohol, smoking, etc) and specific pre-existing comorbidities (eg, prior heart disease, diabetes, hypertension, etc).^27^ These factors may increase susceptibility to air pollution and potentially influence the relationship between air pollution and AMI mortality.^28^, ^29^, ^30^ Thirdly, spatial resolution could not be adjusted to the scale of neighborhoods subject to high levels of pollution. The literature shows that areas located near major roads or industrial zones expose the populations who work or live there to a higher risk of AMI.^31^ Fourth, hypotheses other than air pollution have been put forward to explain the Monday peak. In the absence of a circaseptan biological rhythm, external triggers have generally been considered as possible explanatory theories for the periodic pattern of AMI mortality.^4^ Indeed, the psychological stress associated with returning to work may contributed to the peak in blood pressure and AMI observed on Mondays.^32^, ^33^, ^34^ Last, population studies tend to have lower ORs for AMI events than clinical studies, where each death could be evaluated according to diagnostic criteria.^4^ It is also possible that the number of AMI deaths in this study may have been overestimated because AMI is often considered a “catch-all” cause of death in the absence of a clear medical history or when a confirmatory autopsy is not performed on the deceased.^35^ Nevertheless, previous prospective clinical studies have reported similar Monday peaks, for example, in the incidence of AMI.^2^^,^^36^

## Conclusions

Our study shows a weekly variation in AMI mortality, with a peak on Monday and a minimum on Sunday. Particulate air pollution levels, especially PM_2.5_ and PM_10_, exhibit a similar rhythm, being lower on weekends than on weekdays. Although the highest weekly increase in PM_2.5_ and PM_10_ levels occurs between Sunday and Monday, this does not affect the cyclical pattern of AMI mortality. Adjusting for daily mean pollution levels does not change this pattern, nor does it show any association with it. These results suggest that the significant difference in air pollution between Sunday and Monday is not associated with the cyclical increase in AMI deaths on Mondays observed in the Spanish population.

## Funding support and author disclosures

Drs Achebak, Chen, and Ballester have received funding from the European Union’s Horizon 2020 and Horizon Europe research and innovation programs under grant agreements No 865564 (European Research Council Consolidator Grant EARLY-ADAPT), 101069213 (European Research Council Proof-of-Concept HHS-EWS), and 101123382 (European Research Council Proof-of-Concept FORECAST-AIR). Dr Achebak has received funding from the European Union’s Horizon Europe research and innovation program under grant agreement No 101065876 (MSCA Postdoctoral Fellowship TEMP-MOMO). Dr Ballester has received funding from the Ministry of Science and Innovation (MCIU) under grant agreement No RYC2018-025446-I (program Ramón y Cajal). ISGlobal authors acknowledge support from the grant CEX2023-0001290-S funded by MCIN/AEI/10.13039/501100011033, and support from the Generalitat de Catalunya through the CERCA Program. All other authors have reported that they have no relationships relevant to the contents of this paper to disclose.
