# Supplementary material for: Association Between Air Pollution and Monday Peak Mortality From Acute Myocardial Infarction
Source: JACC Adv. 2025 Dec 17;5(1):102378. doi: 10.1016/j.jacadv.2025.102378 (PMC12869875; doi:10.1016/j.jacadv.2025.102378)
Supplement: Supplemental Tables 1 to 3 and Supplemental Figures 1 to 8 [file mmc1.docx]

***Supplemental Material***

**Air pollution and Monday peak in mortality from acute myocardial infarction**

*Ruben Lévy^1-2^, Laurent Lévy^1^, Joan Ballester^3^, Zhao-Yue Chen^3^, François R Herrmann^1-2^, Antonio Gasparrini^4^, Hicham Achebak^3-5^*

^1^Medical School of the University of Geneva, Geneva, Switzerland; ^2^Division of Geriatrics, Department of Rehabilitation and Geriatrics, Geneva University Hospitals, Thônex, Switzerland; ^3^ISGlobal, Barcelona, Spain; ^4^Environment & Health Modelling (EHM) Lab, Department of Public Health Environments and Society, London School of Hygiene & Tropical Medicine (LSHTM), United Kingdom; ^5^Inserm, France Cohortes, Paris, France.

Correspondence to:

Dr. Hicham Achebak,

Inserm, France Cohortes,

75013 Paris, France,

**hicham.achebak@inserm.fr**

**Table of contents**

[Supplemental Figure 1. Map of the Spanish provinces 3](#_Toc209529896)

[Supplemental Figure 2. Evolution of mortality and air pollution 4](#_Toc209529897)

[Supplemental Figure 3. Weekly variation of mortality from different causes of disease 5](#_Toc209529898)

[Supplemental Figure 4. Weekly variation in AMI mortality before and after adjustment for lagged daily air pollution 6](#_Toc209529899)

[Supplemental Figure 5. Weekly variation in AMI mortality before and after adjustment for daily air pollution by season 8](#_Toc209529900)

[Supplemental Figure 6. Weekly variation in AMI mortality before and after adjustment for inter-day change in pollution by season 10](#_Toc209529901)

[Supplemental Figure 7. Weekly variation in AMI mortality before and after adjustment for daily air pollution by degree of urbanisation 12](#_Toc209529902)

[Supplemental Figure 8. Weekly variation in AMI mortality before and after adjustment for inter-day change in pollution by degree of urbanisation 14](#_Toc209529903)

[Supplemental Table 1. Monday excess in AMI mortality risk by degree of urbanisation 16](#_Toc209529904)

[Supplemental Table 2. Analysis of heterogeneity from multivariate meta-analysis of the weekly variation in AMI mortality 17](#_Toc209529905)

[Supplemental Table 3. Analysis of heterogeneity from meta-analysis the association between air pollution and Monday peak in AMI 18](#_Toc209529906)

# **Supplemental Figure 1. Map of the Spanish provinces**

|  |
| --- |
| 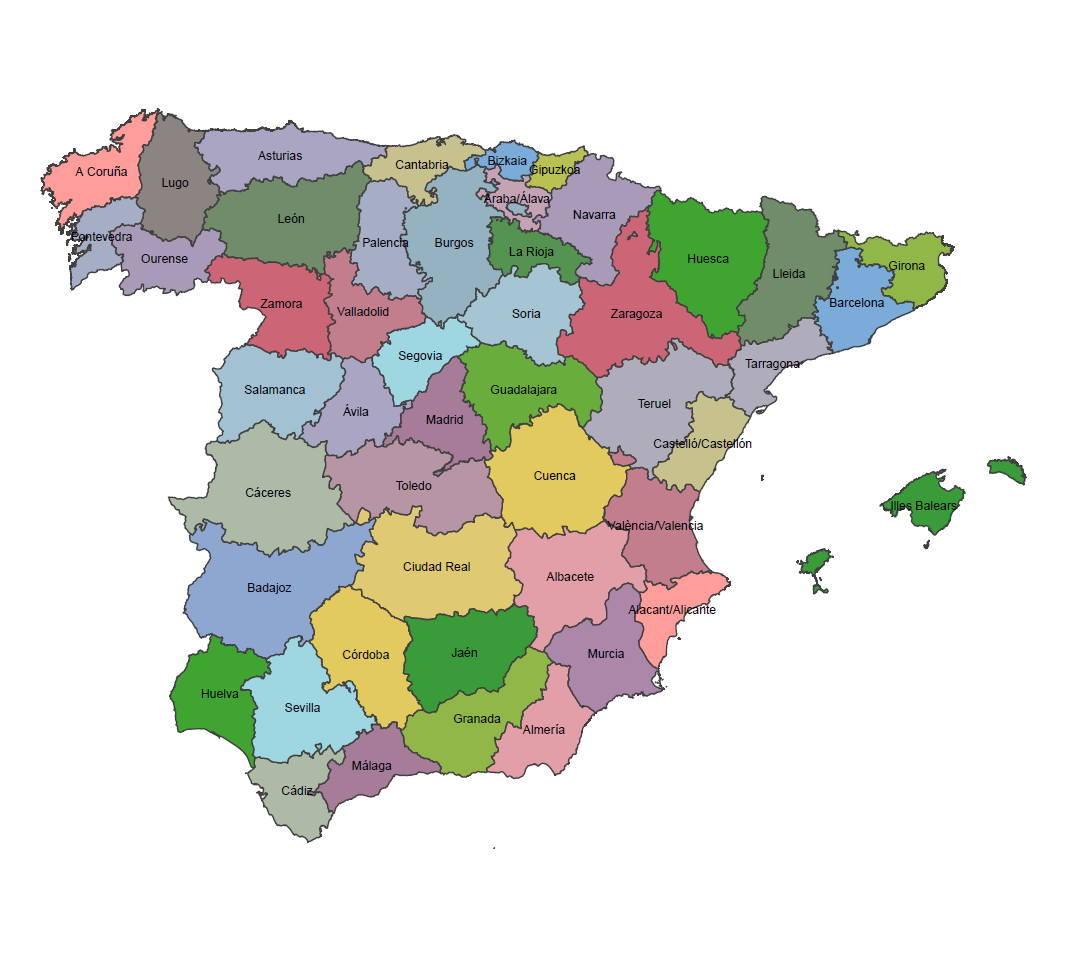 |
|  |

# **Supplemental Figure 2. Evolution of mortality and air pollution**

AMI=Acute myocardial infarction. IHD=Ischemic heart diseases.

|  |  |
| --- | --- |
| a) Raw mortality rate | b) PM_2.5_ |
|  |  |
| 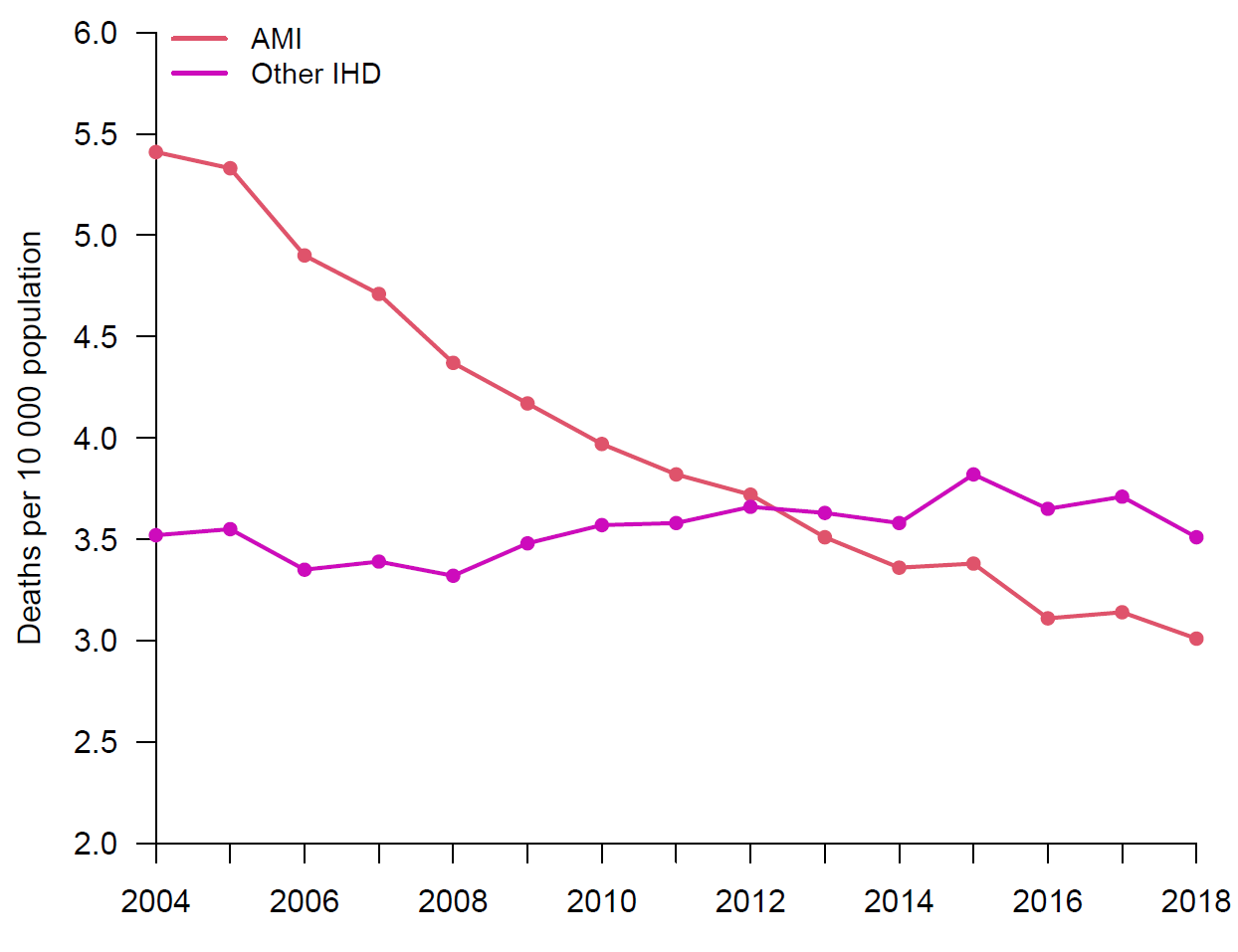 | 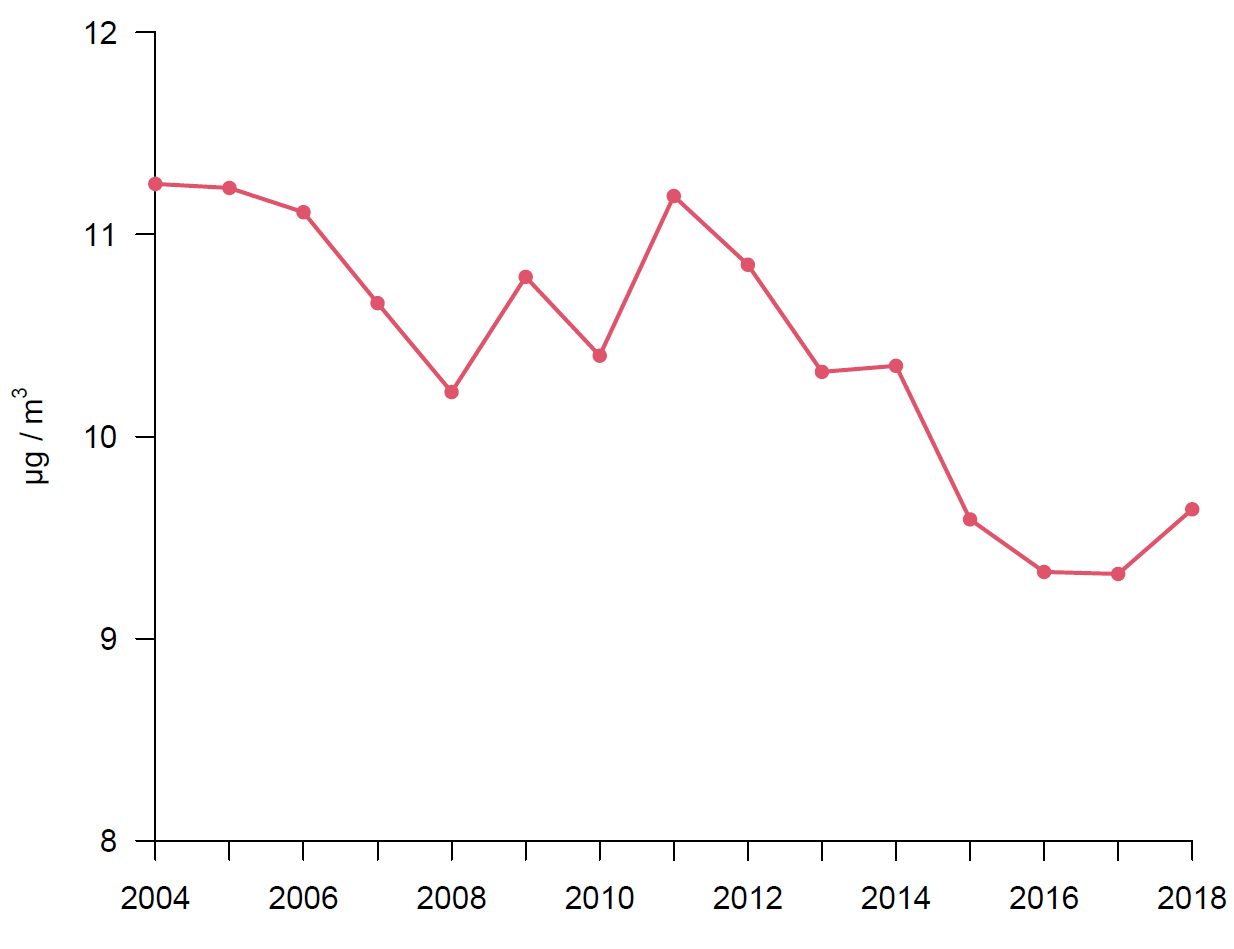 |
|  |  |
| c) PM_10_ | d) NO_2_ |
|  |  |
| 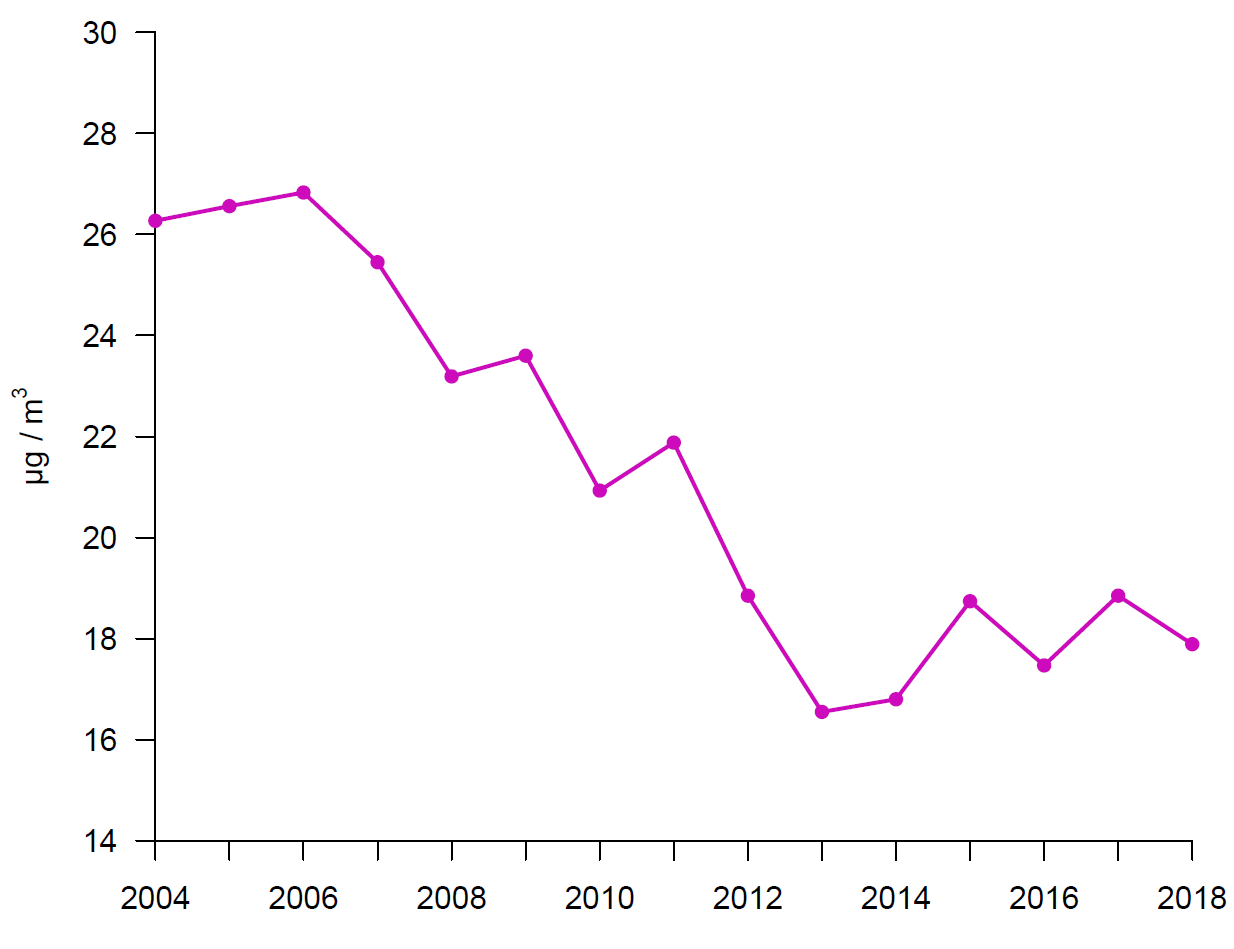 | 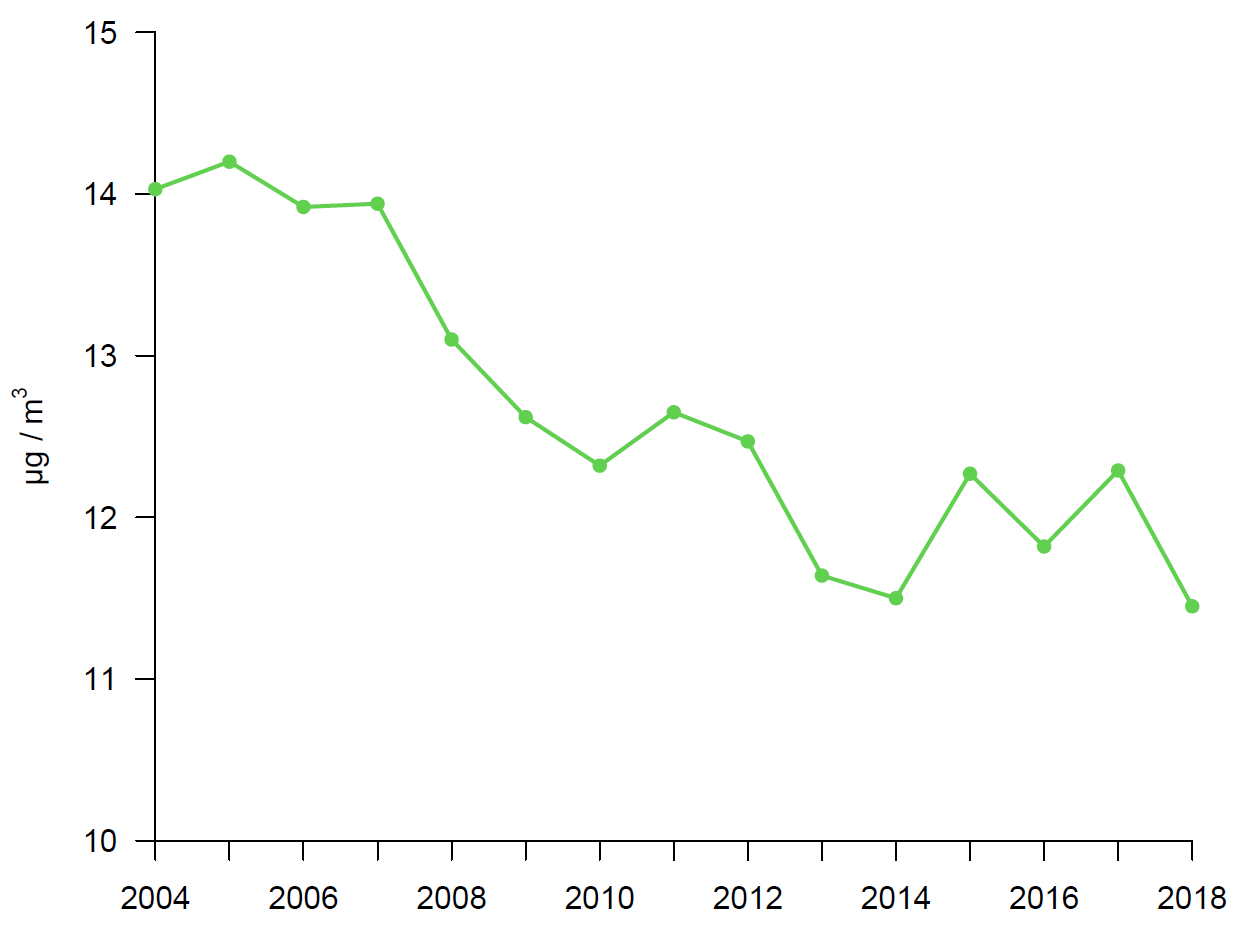 |
|  |  |
| e) O_3_ |  |
|  |  |
| 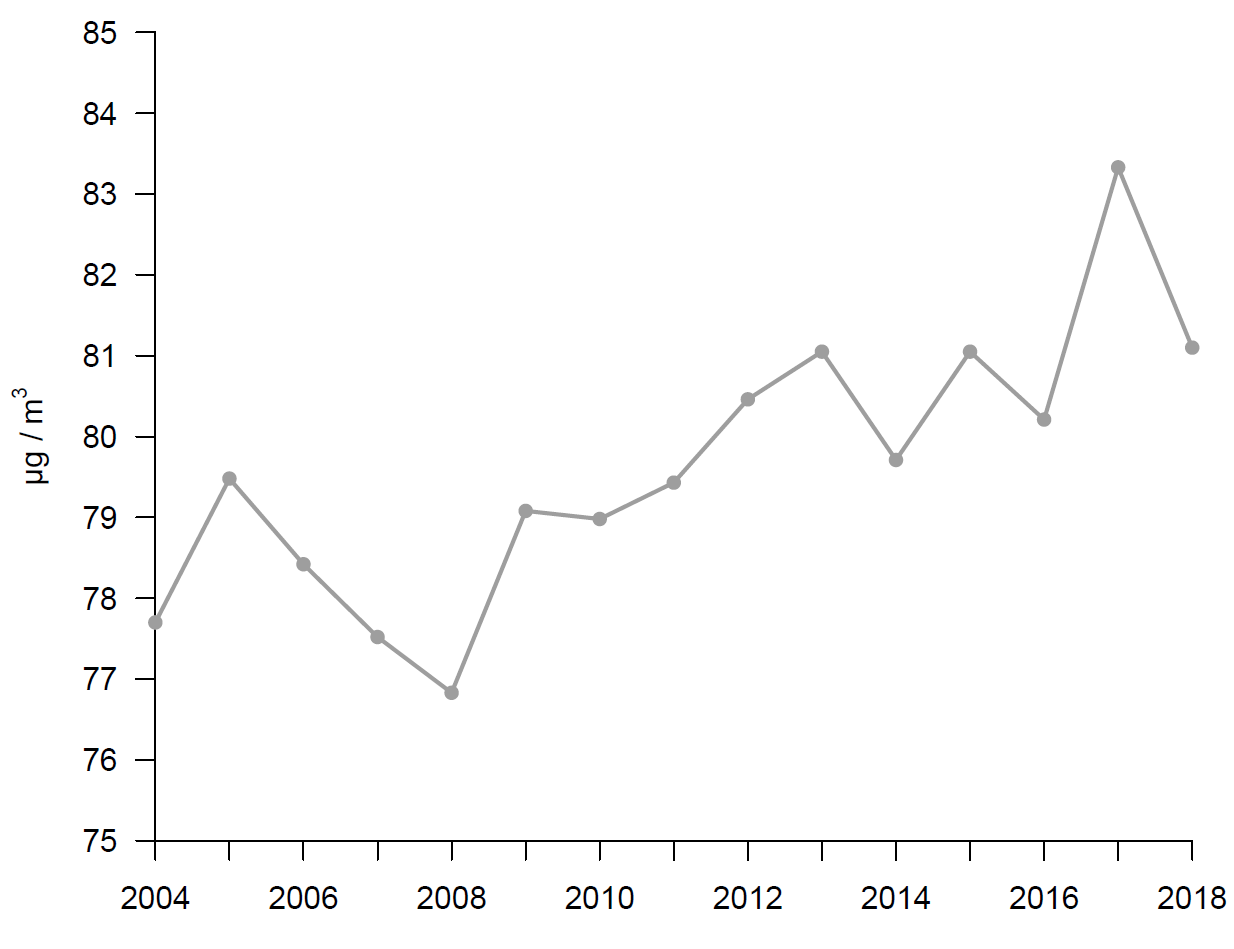 |  |
|  |  |

# **Supplemental Figure 3.** **Weekly variation of mortality from different causes of disease**

The values represent the percent change in deaths with regard to the weekly average.

|  |
| --- |
| a) |
|  |
| 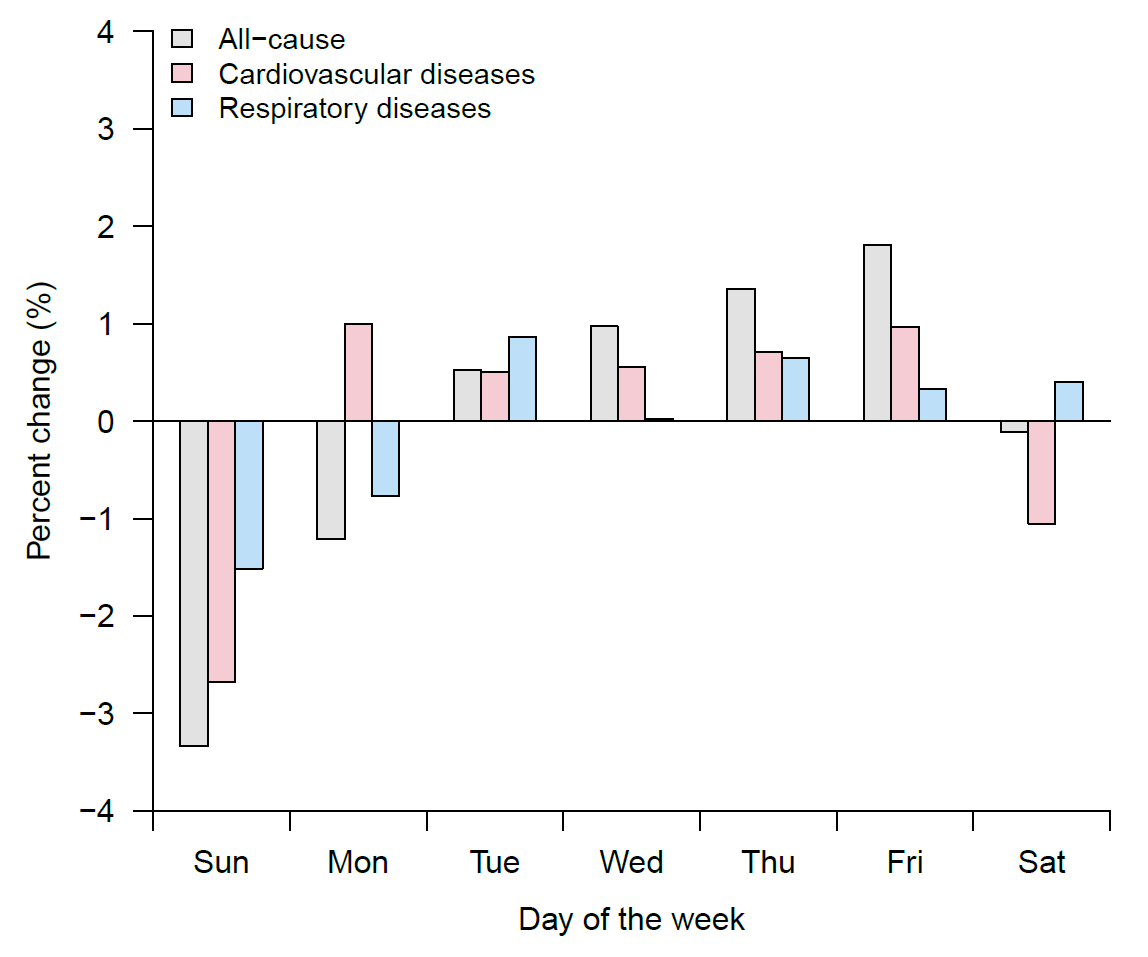 |
|  |
| b) |
|  |
| 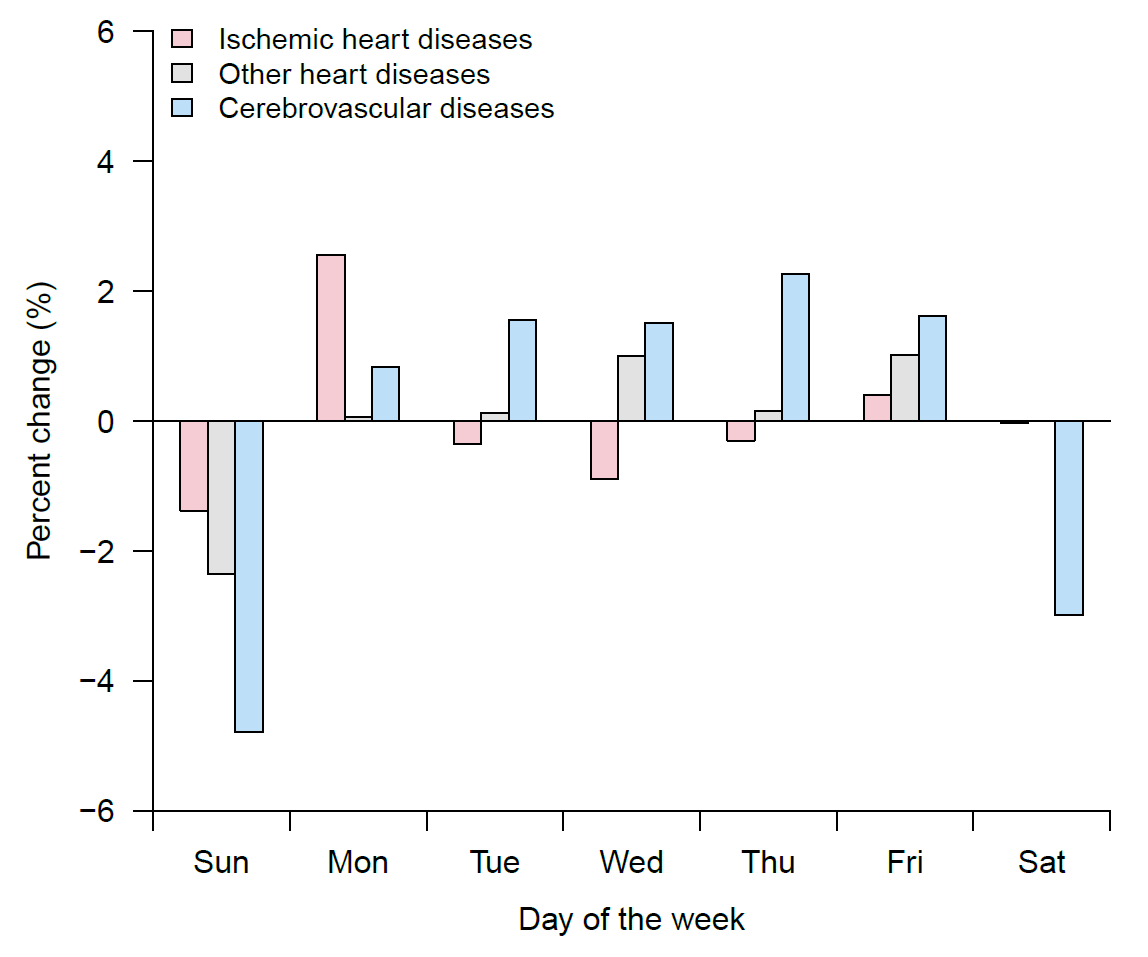 |
|  |
| c) |
|  |
| 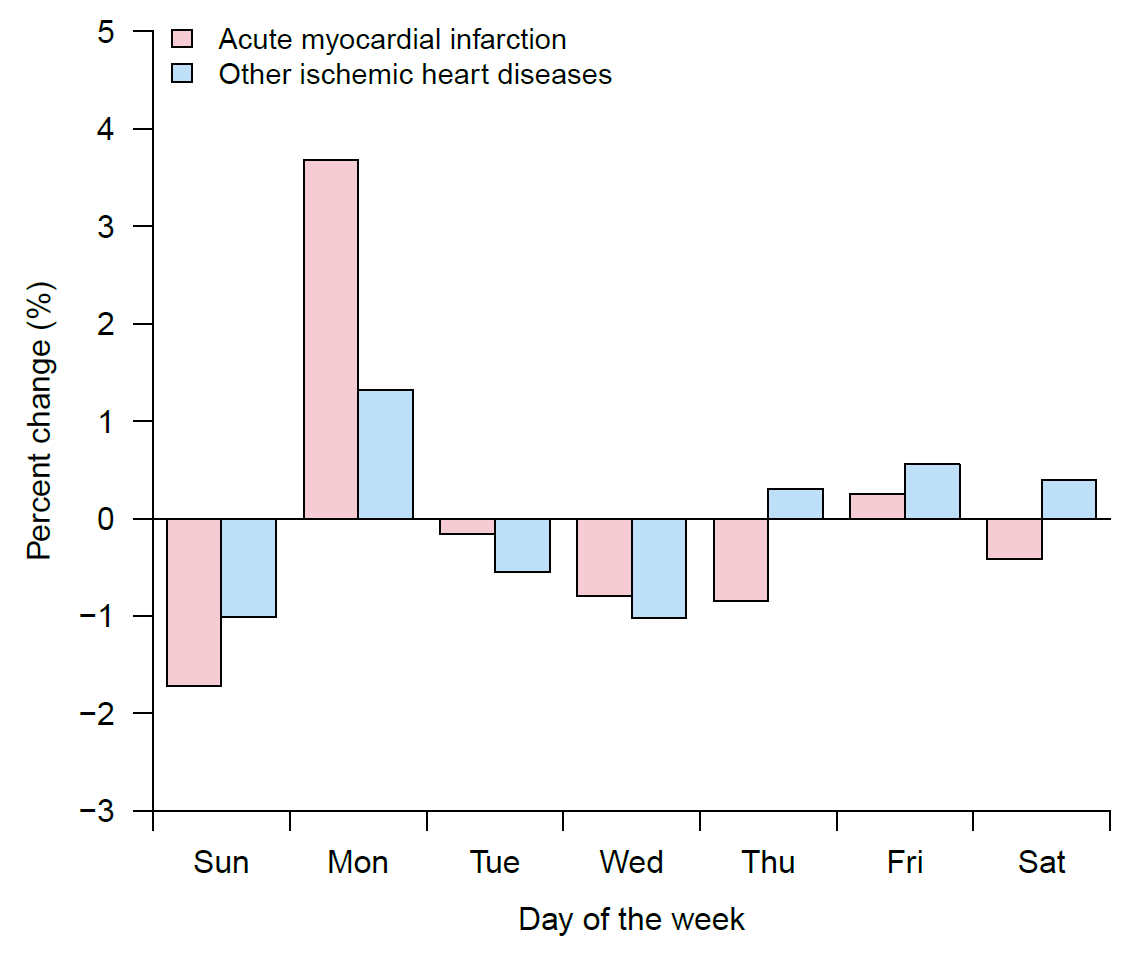 |
|  |

# **Supplemental Figure 4. Weekly variation in AMI mortality before and after adjustment for lagged daily air pollution**

RR=relative risk. RR curves are computed using Sunday as a reference. Winter=December-March, Summer=June-September.

|  |  |
| --- | --- |
| **1. Adjusted by daily mean PM_2·5_ concentration** | |
|  |  |
| a) Lag 1 | b) Lag 2 |
|  |  |
| 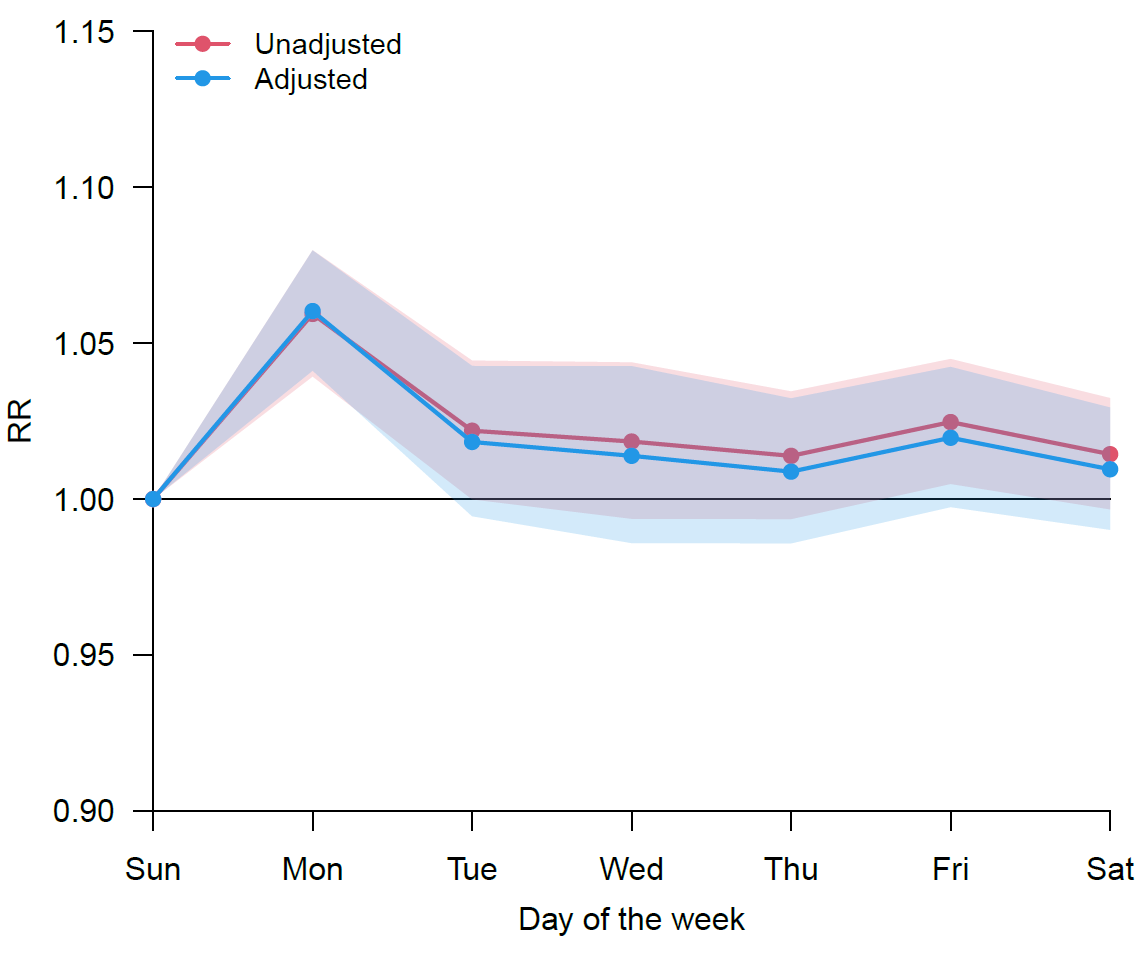 | 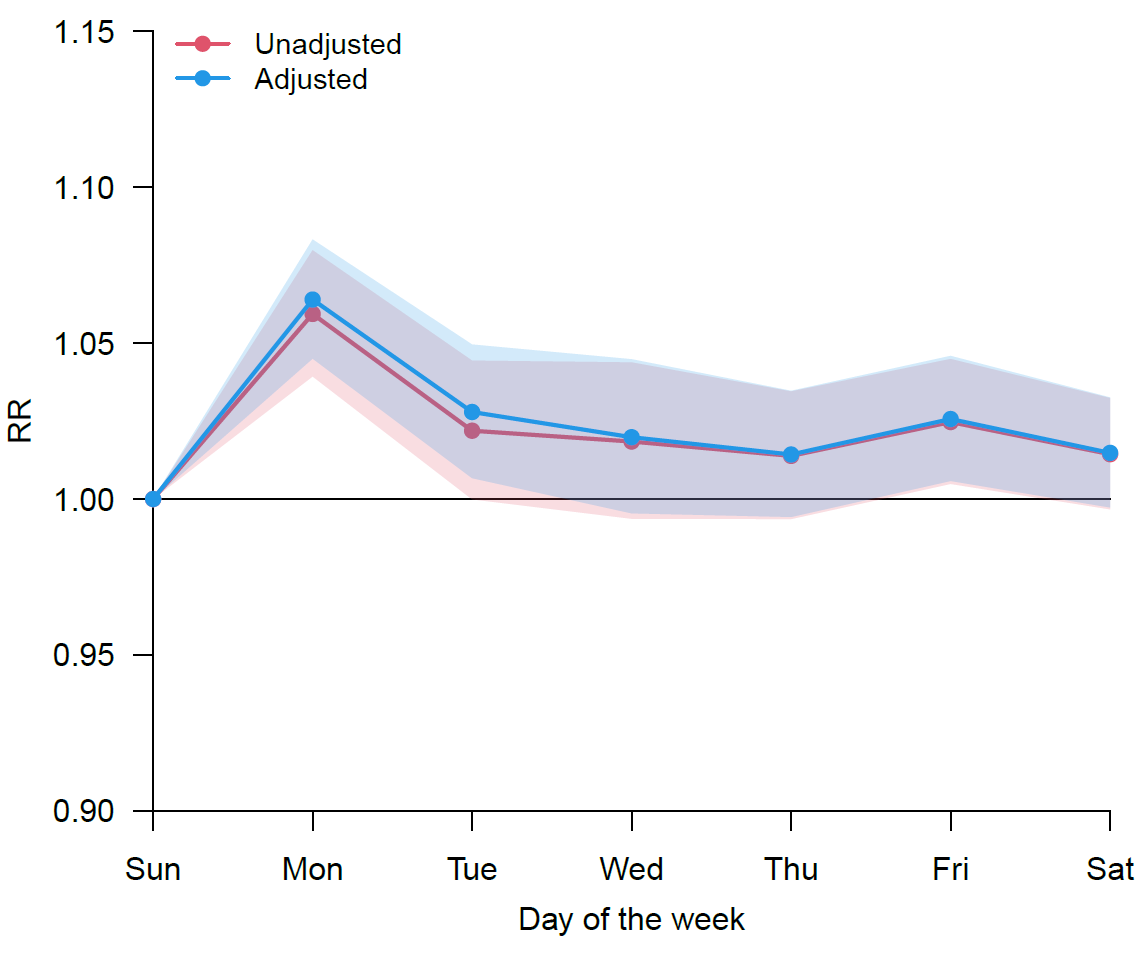 |
|  |  |
| **2. Adjusted by daily mean PM_10_ concentration** | |
|  |  |
| c) Lag 1 | d) Lag 2 |
|  | |
| 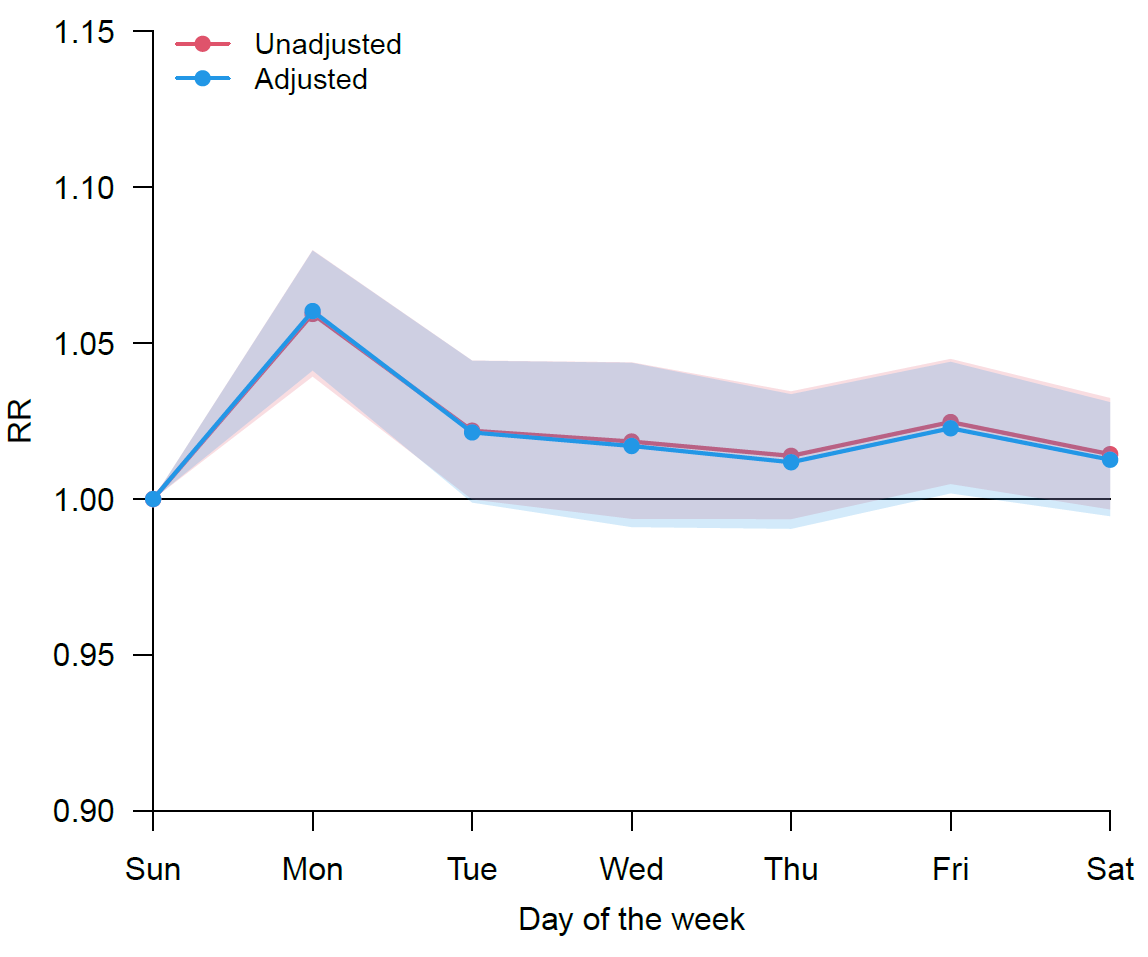 | 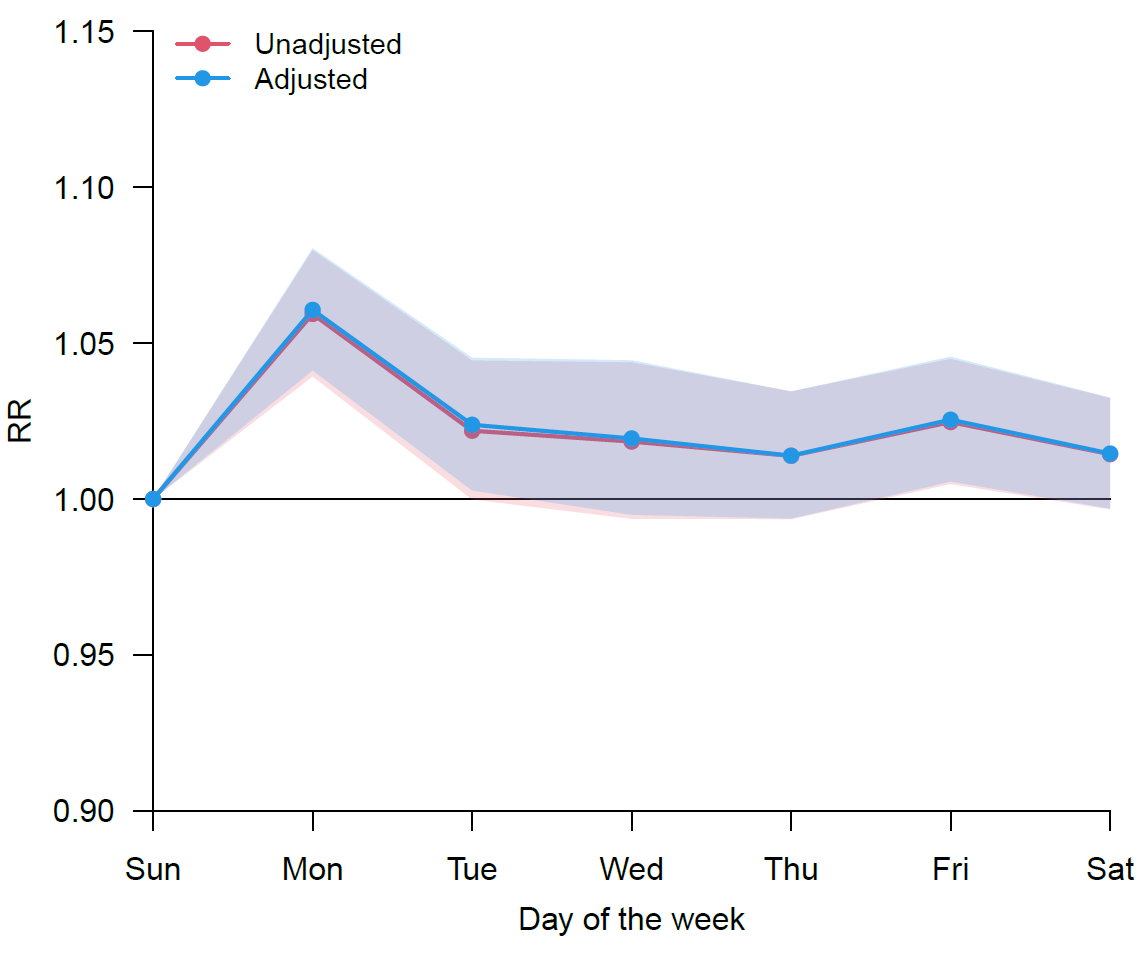 |
|  |  |
| **3. Adjusted by daily mean NO_2_ concentration** | |
|  |  |
| e) Lag 1 | f) Lag 2 |
|  |  |
| 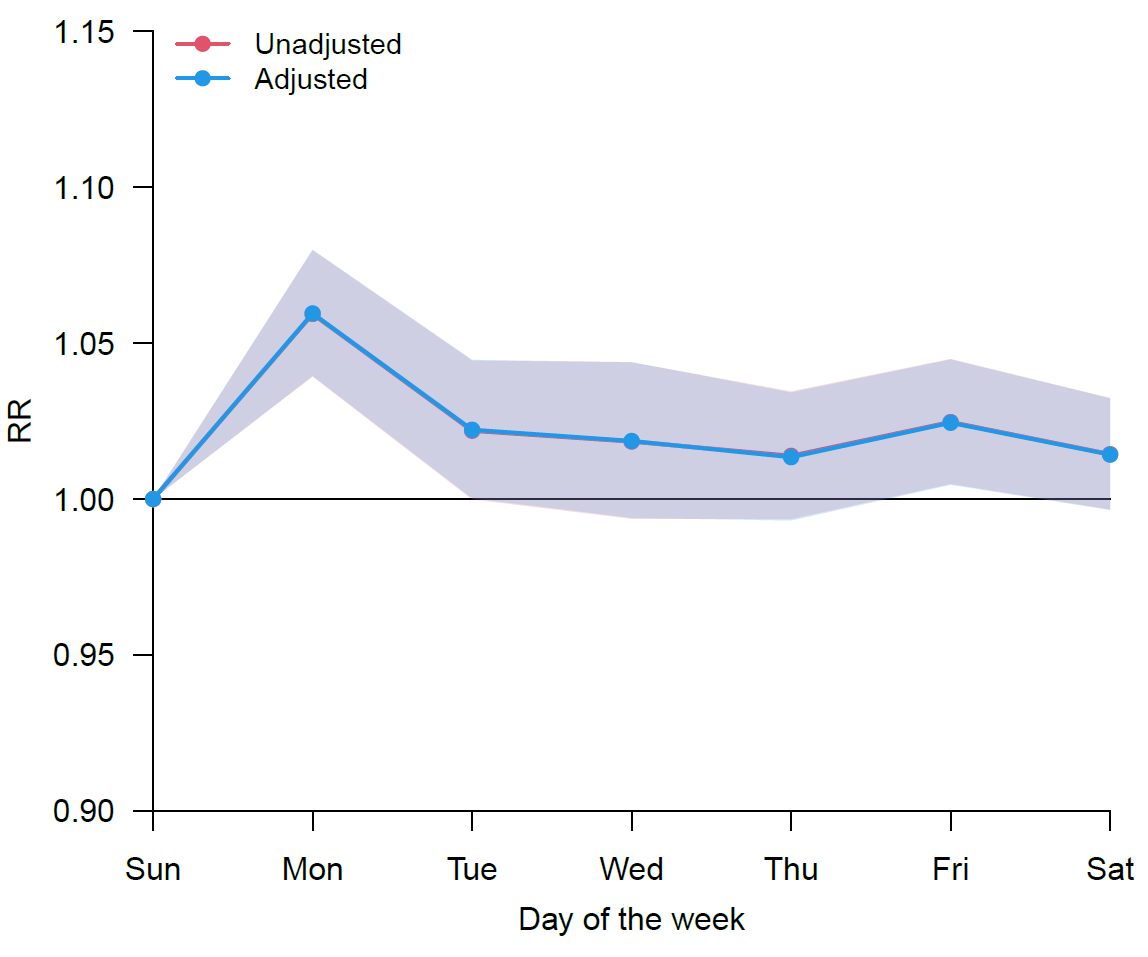 | 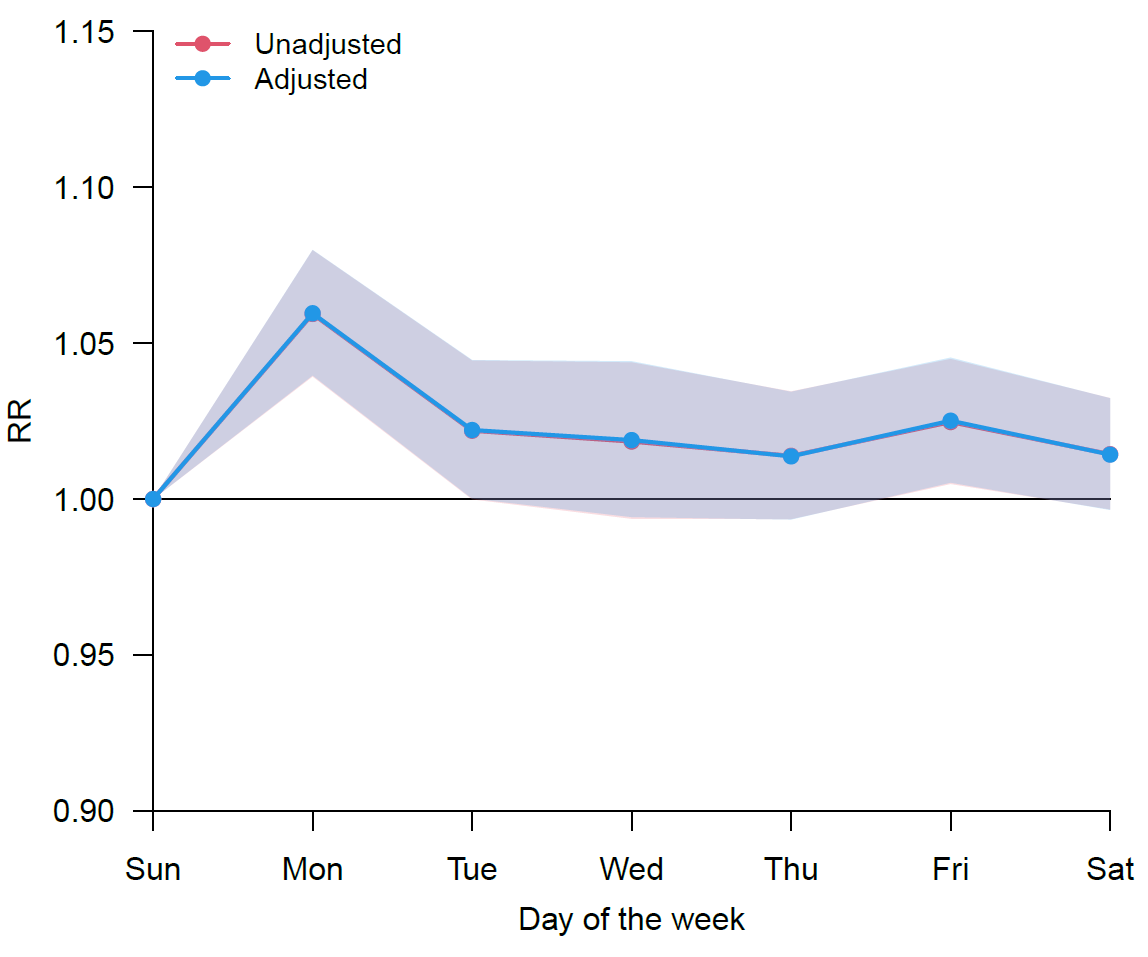 |
|  |  |
| **4. Adjusted by daily mean O_3_ concentration** | |
|  |  |
| g) Lag 1 | h) Lag 2 |
|  |  |
| 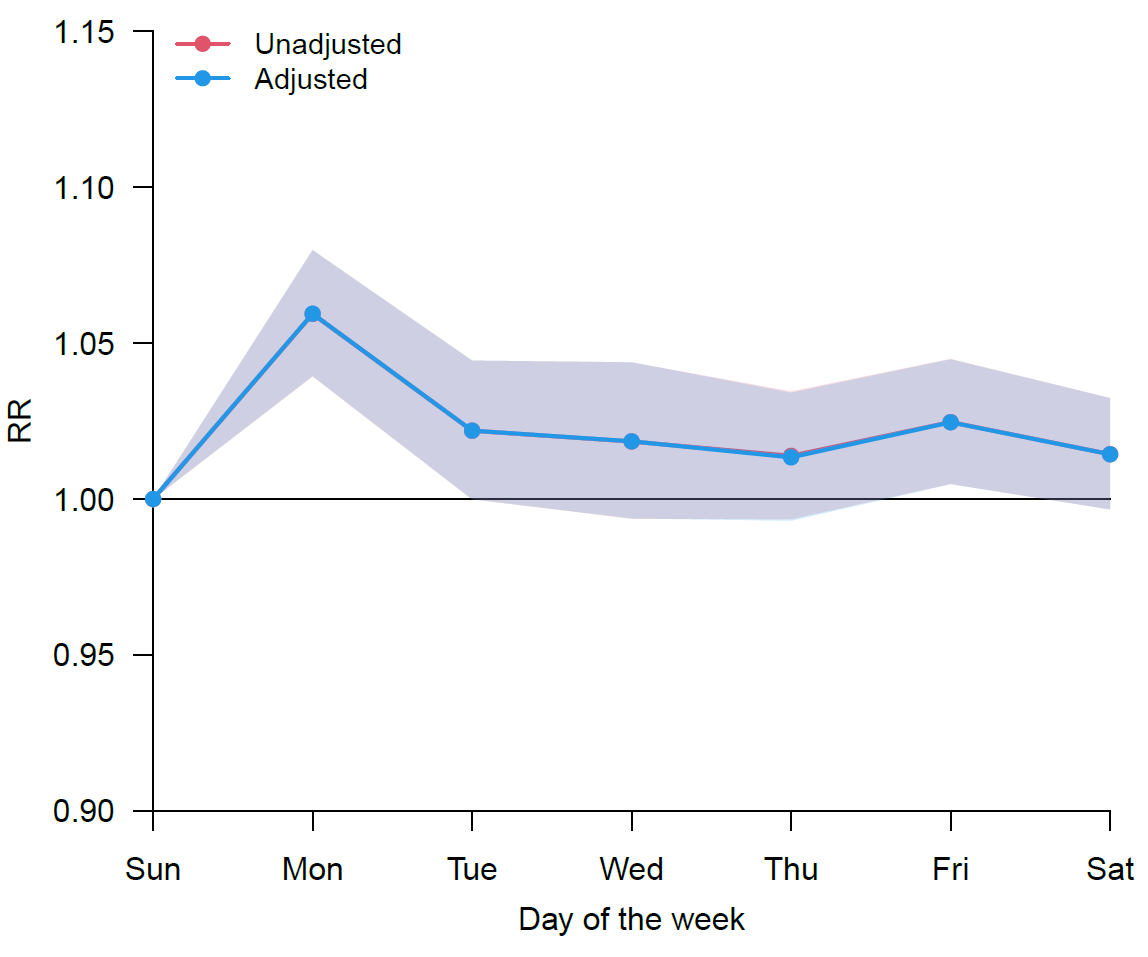 | 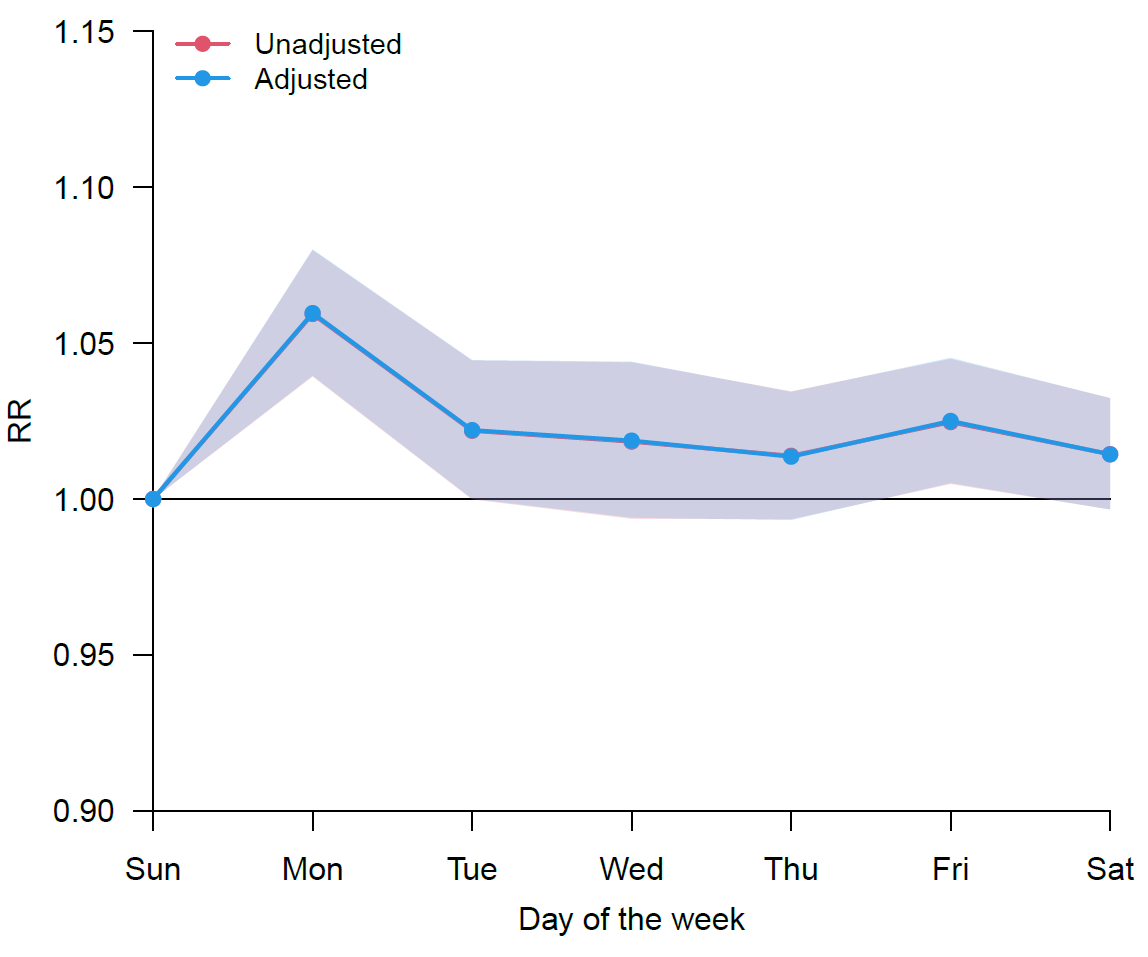 |
|  |  |

# **Supplemental Figure 5.** **Weekly variation in AMI mortality before and after adjustment for daily air pollution by season**

RR=relative risk. RR curves are computed using Sunday as a reference. Winter=December-March, Summer=June-September.

|  |  |
| --- | --- |
| **1. Adjusted by daily mean PM_2·5_ concentration** | |
|  |  |
| a) Winter | b) Summer |
|  |  |
| 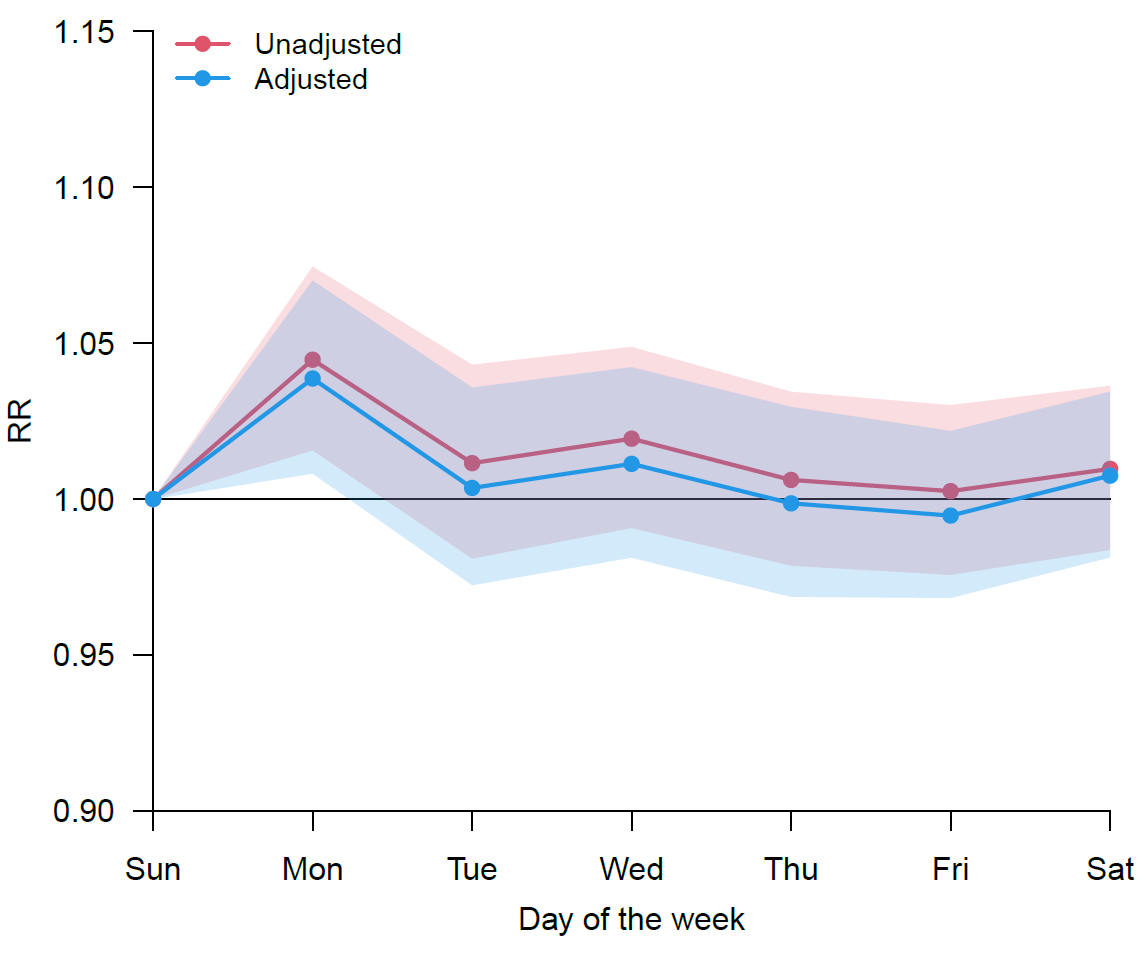 | 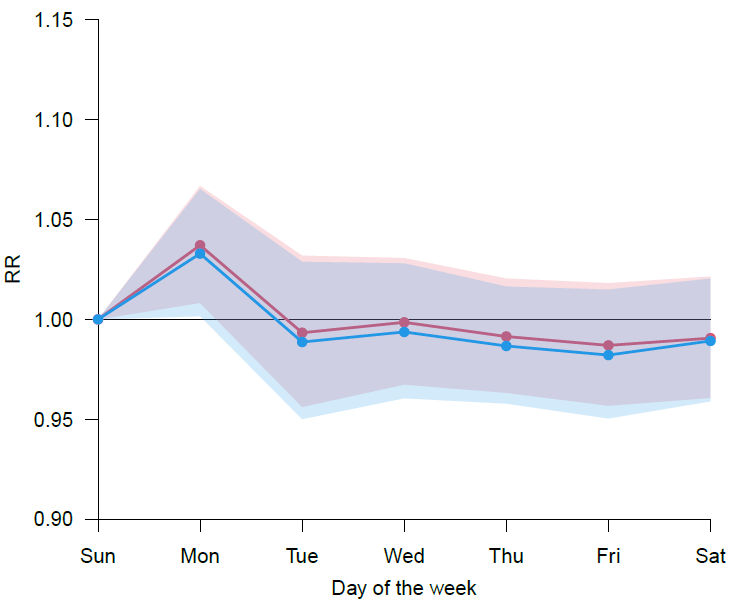 |
|  |  |
| **2. Adjusted by daily mean PM_10_ concentration** | |
|  |  |
| c) Winter | d) Summer |
|  | |
| 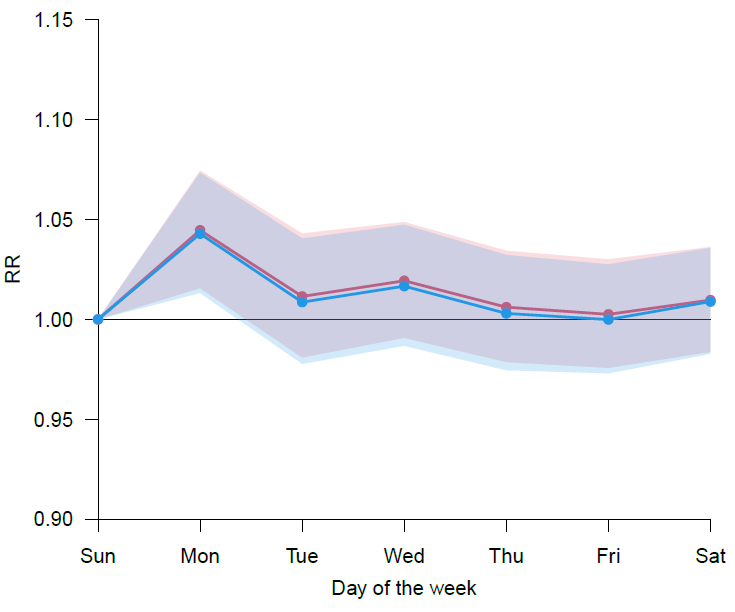 | 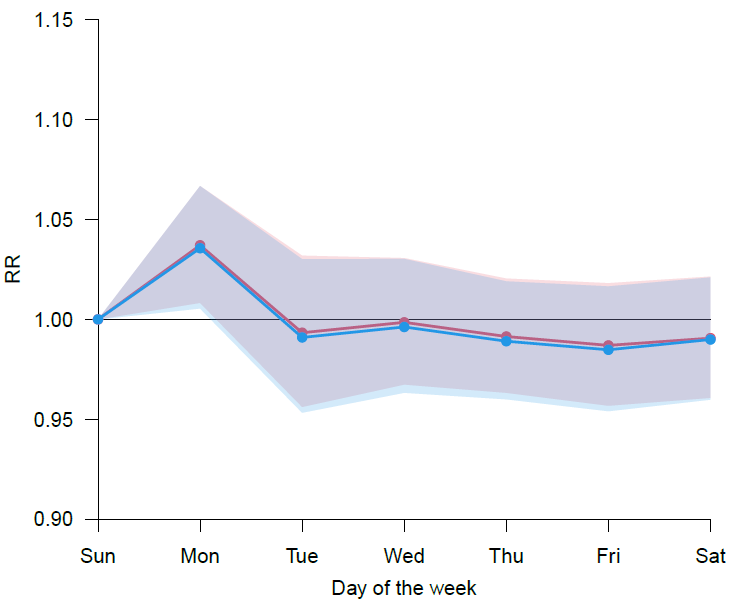 |
|  |  |
| **3. Adjusted by daily mean NO_2_ concentration** | |
|  |  |
| e) Winter | f) Summer |
|  |  |
| 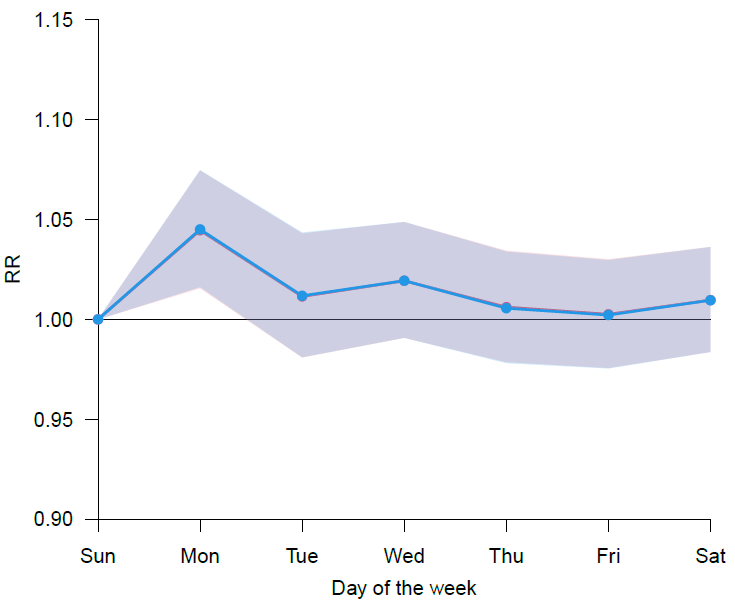 | 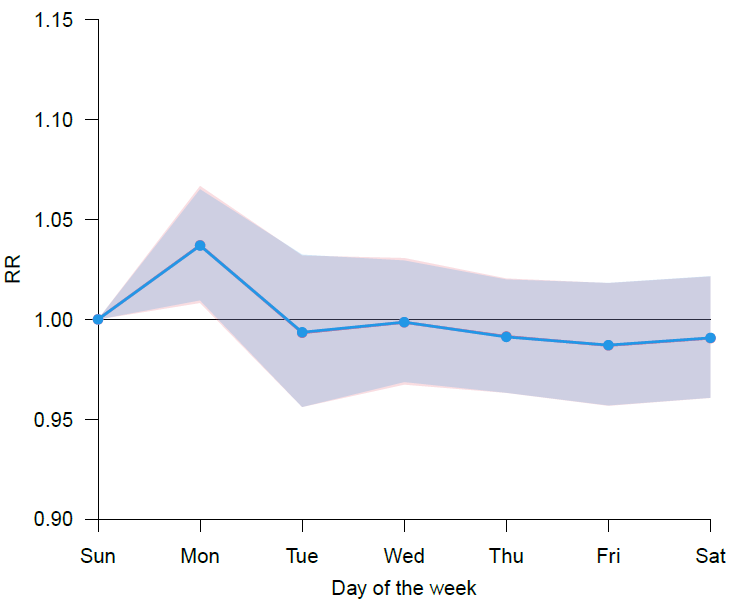 |
|  |  |
| **4. Adjusted by daily mean O_3_ concentration** | |
|  |  |
| g) Winter | h) Summer |
|  |  |
| 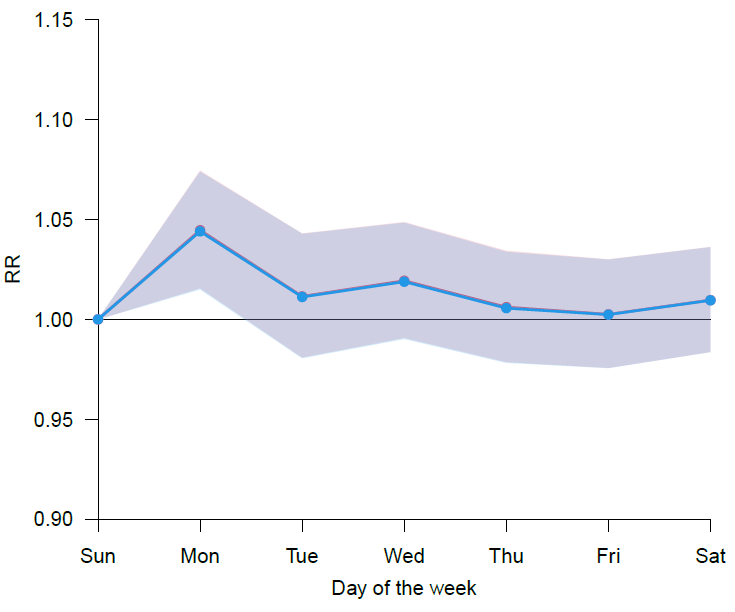 | 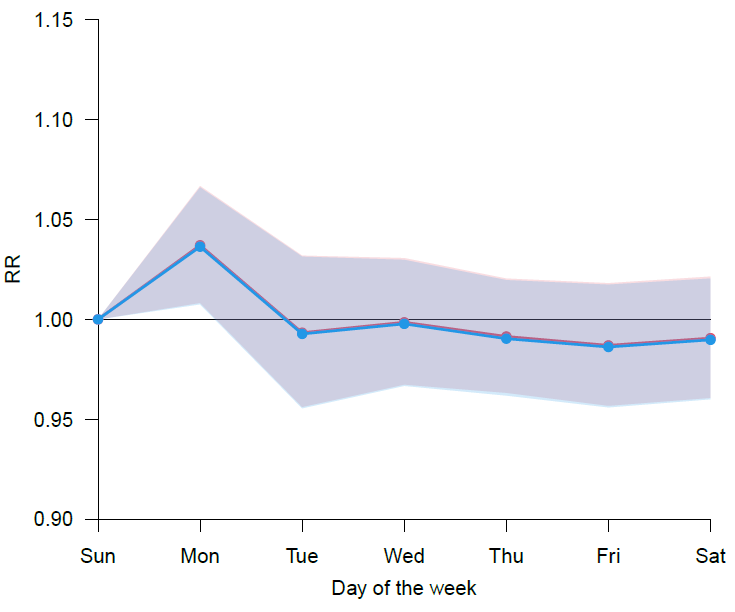 |
|  |  |

# **Supplemental Figure 6. Weekly variation in AMI mortality before and after adjustment for inter-day change in pollution by season**

RR=relative risk. RR curves are computed using Sunday as a reference. Winter=December-March, Summer=June-September.

|  |  |
| --- | --- |
| **1. Adjusted by inter-day change in PM_2·5_ concentration** | |
|  |  |
| a) Winter | b) Summer |
|  |  |
| 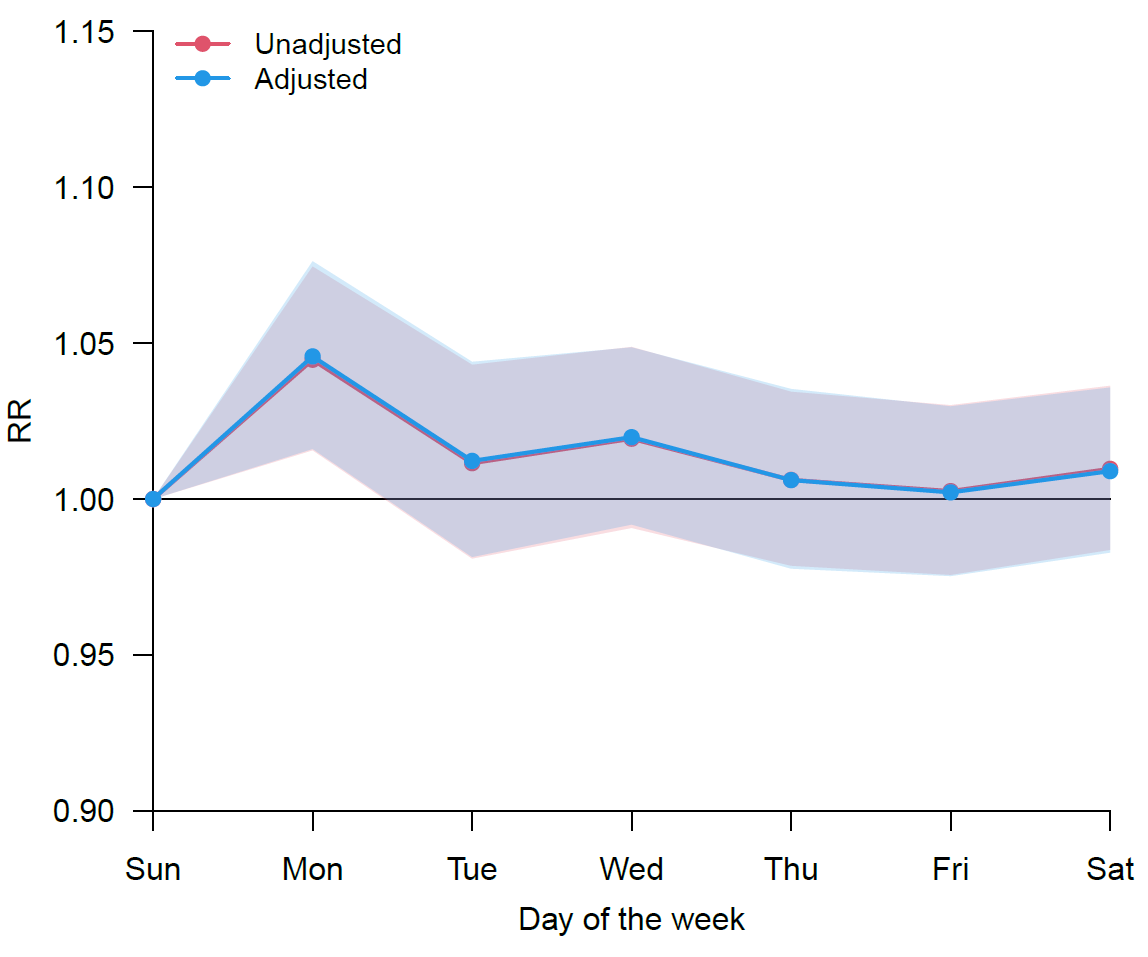 | 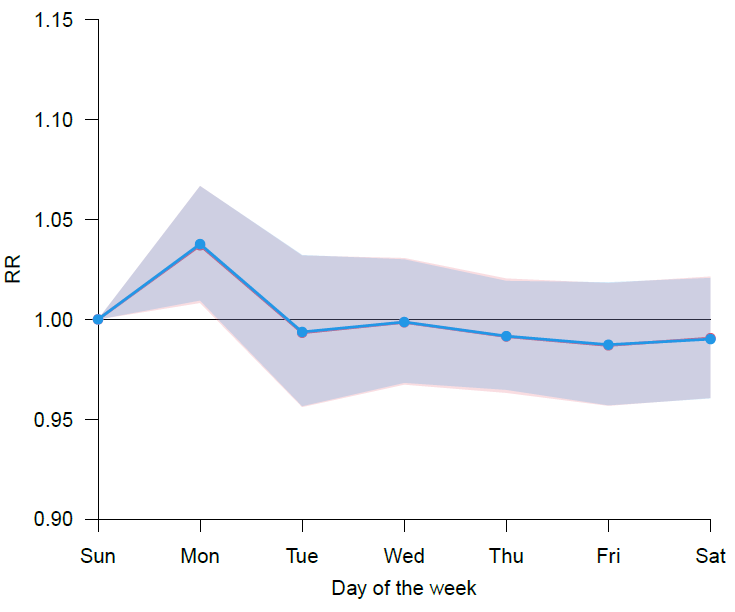 |
|  |  |
| **2. Adjusted by inter-day change in PM_10_ concentration** | |
|  |  |
| c) Winter | d) Summer |
|  | |
| 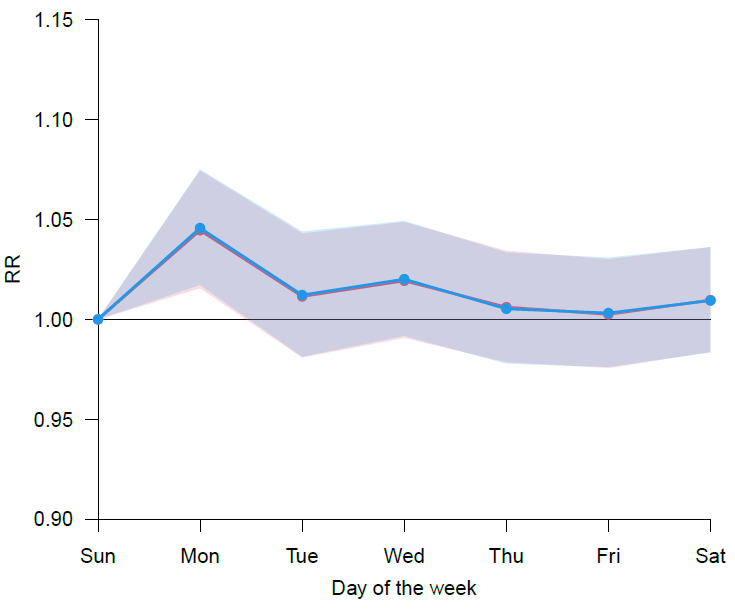 | 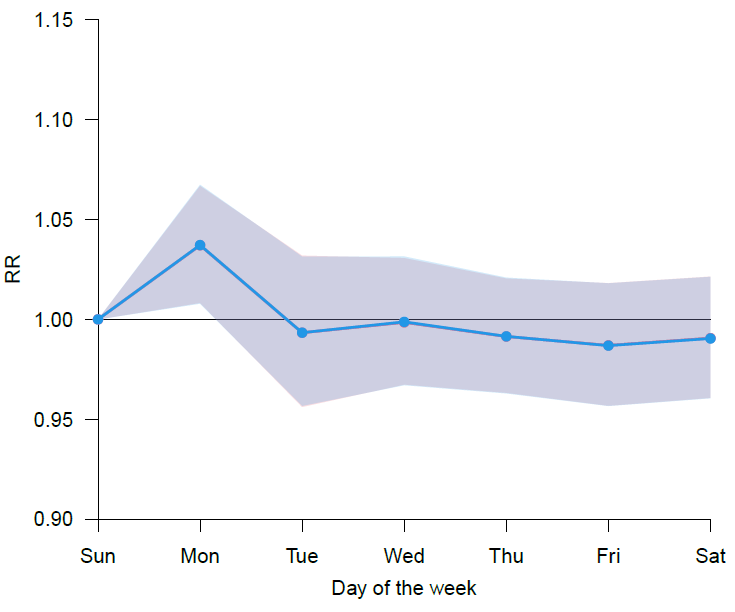 |
|  |  |
| **3. Adjusted by inter-day change in NO_2_ concentration** | |
|  |  |
| e) Winter | f) Summer |
|  |  |
| 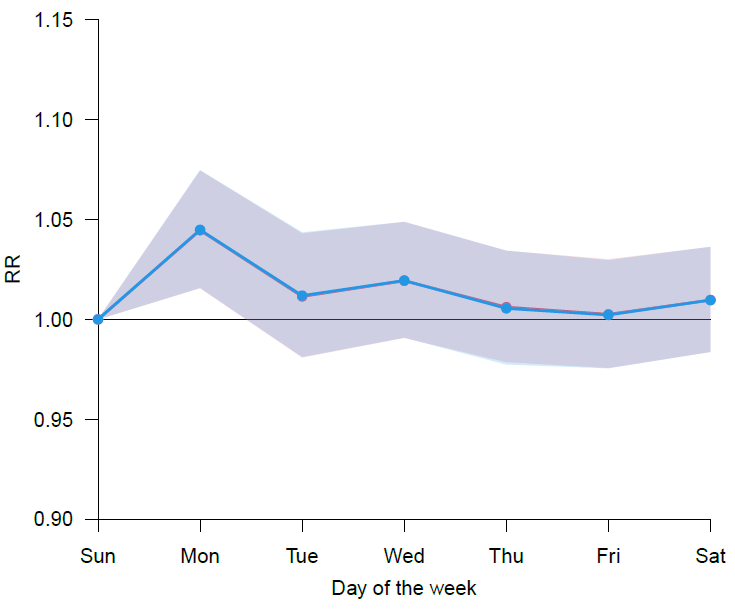 | 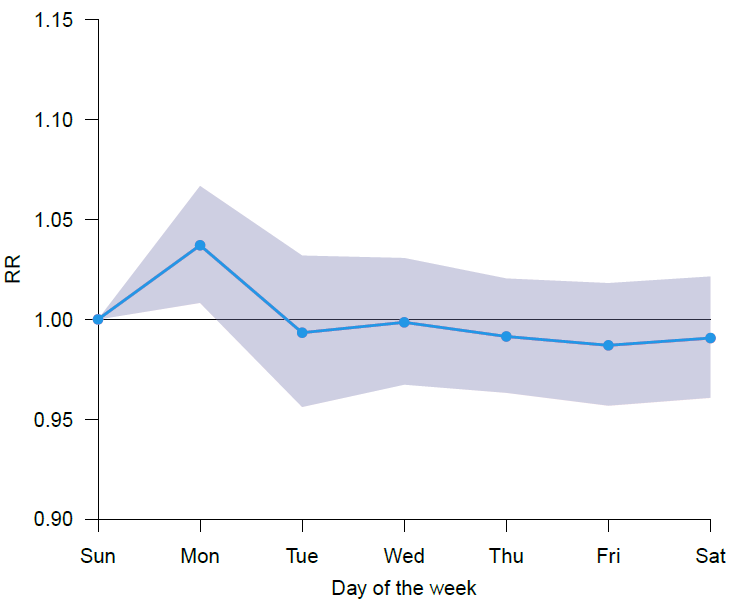 |
|  |  |
| **4. Adjusted by inter-day change in O_3_ concentration** | |
|  |  |
| g) Winter | h) Summer |
|  |  |
| 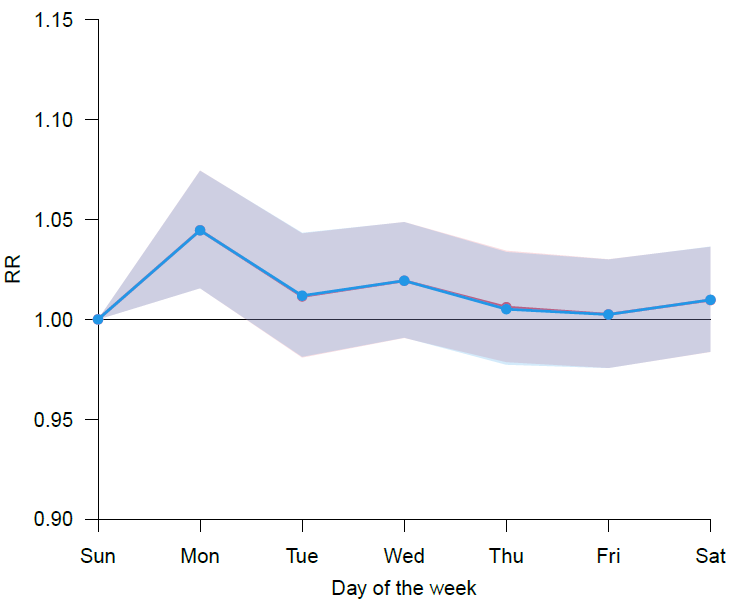 | 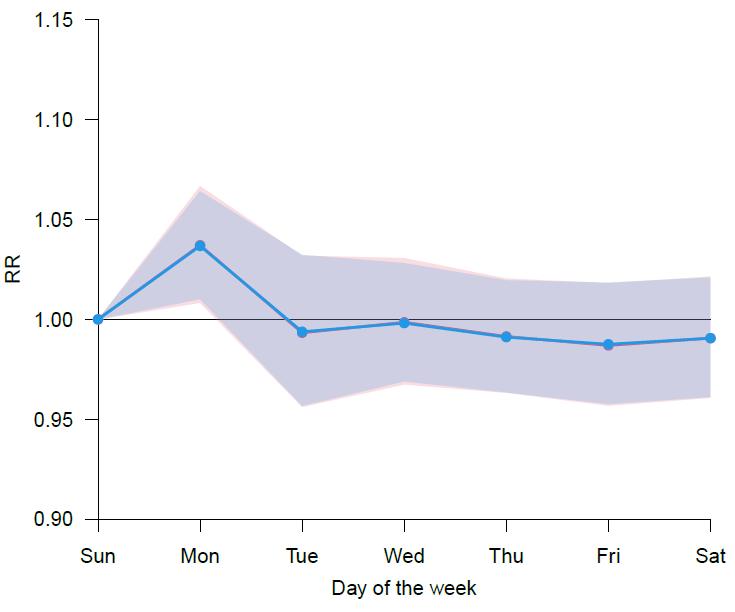 |
|  |  |

# **Supplemental Figure 7. Weekly variation in AMI mortality before and after adjustment for daily air pollution by degree of urbanisation**

RR=relative risk. RR curves are computed using Sunday as a reference. Q=Quartile. Quartiles are based on population density data.

|  |  |  |  |
| --- | --- | --- | --- |
| **1. Adjusted by daily mean PM_2·5_ concentration** | |  |  |
|  |  |  |  |
| a) Q1 | b) Q2 | c) Q3 | d) Q4 |
|  |  |  |  |
| 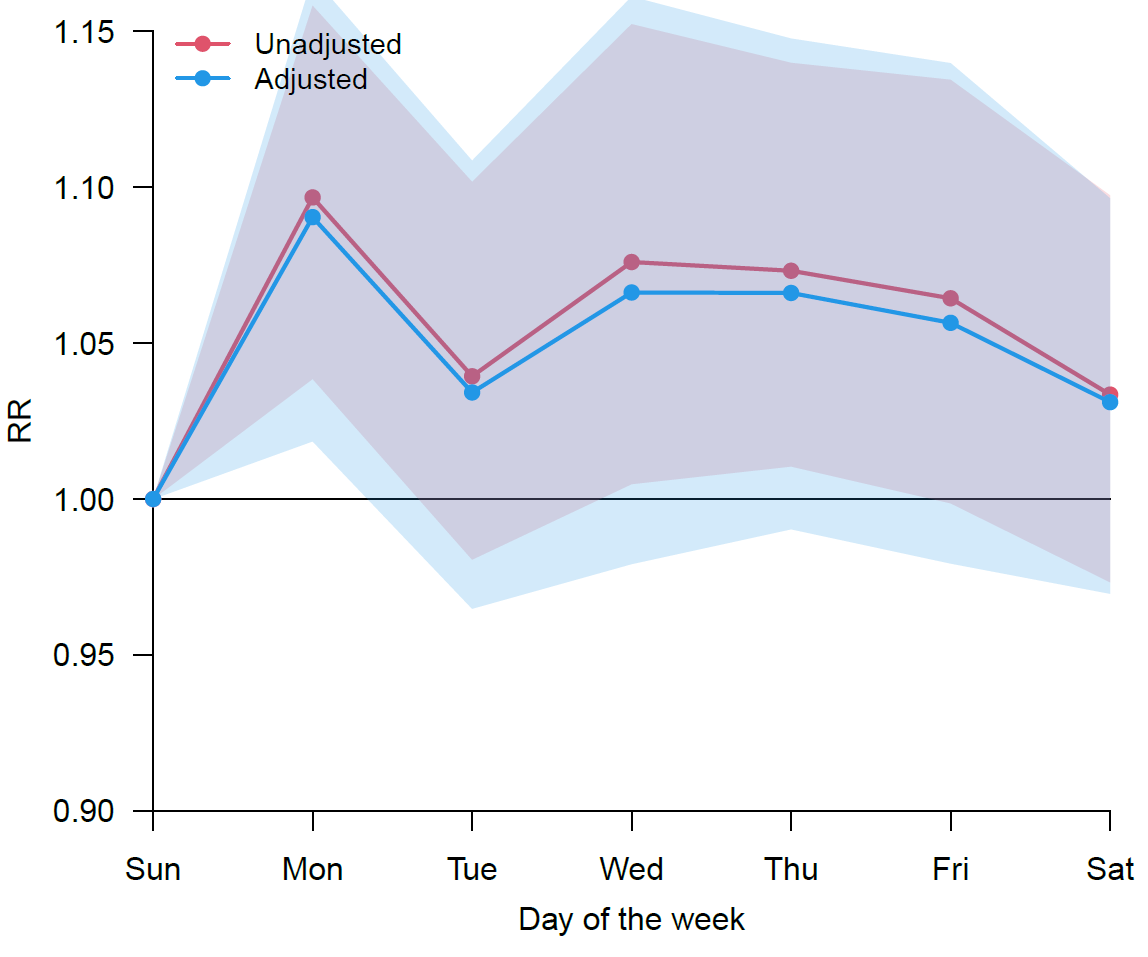 | 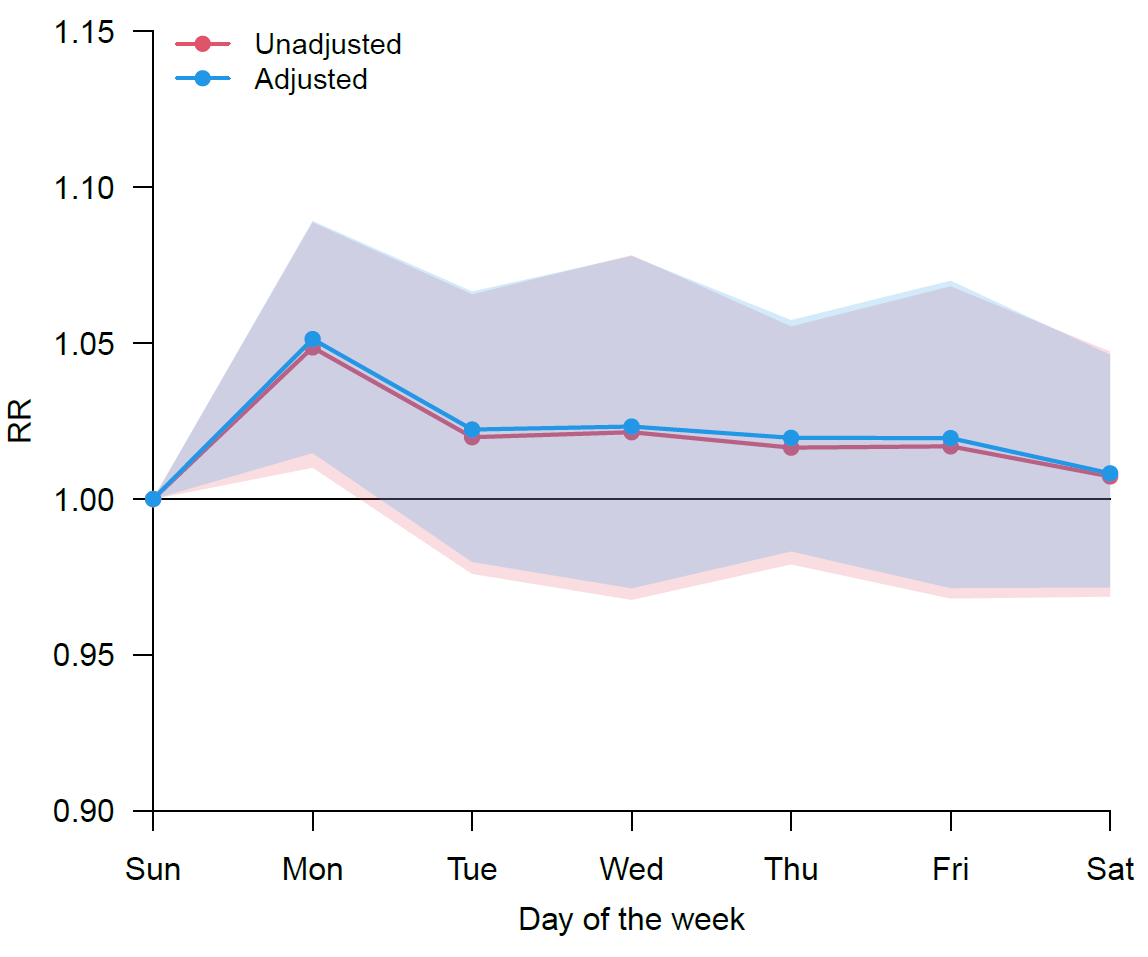 | 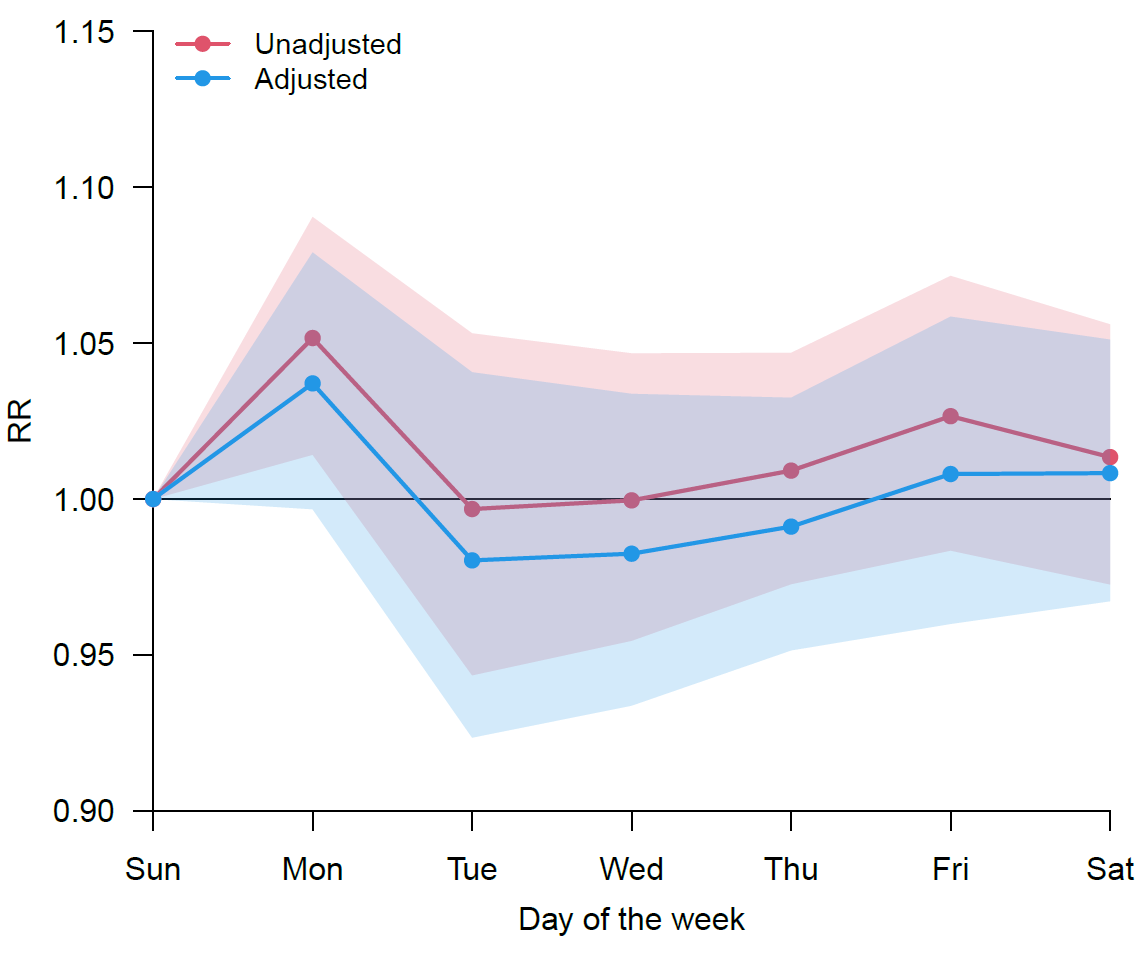 | 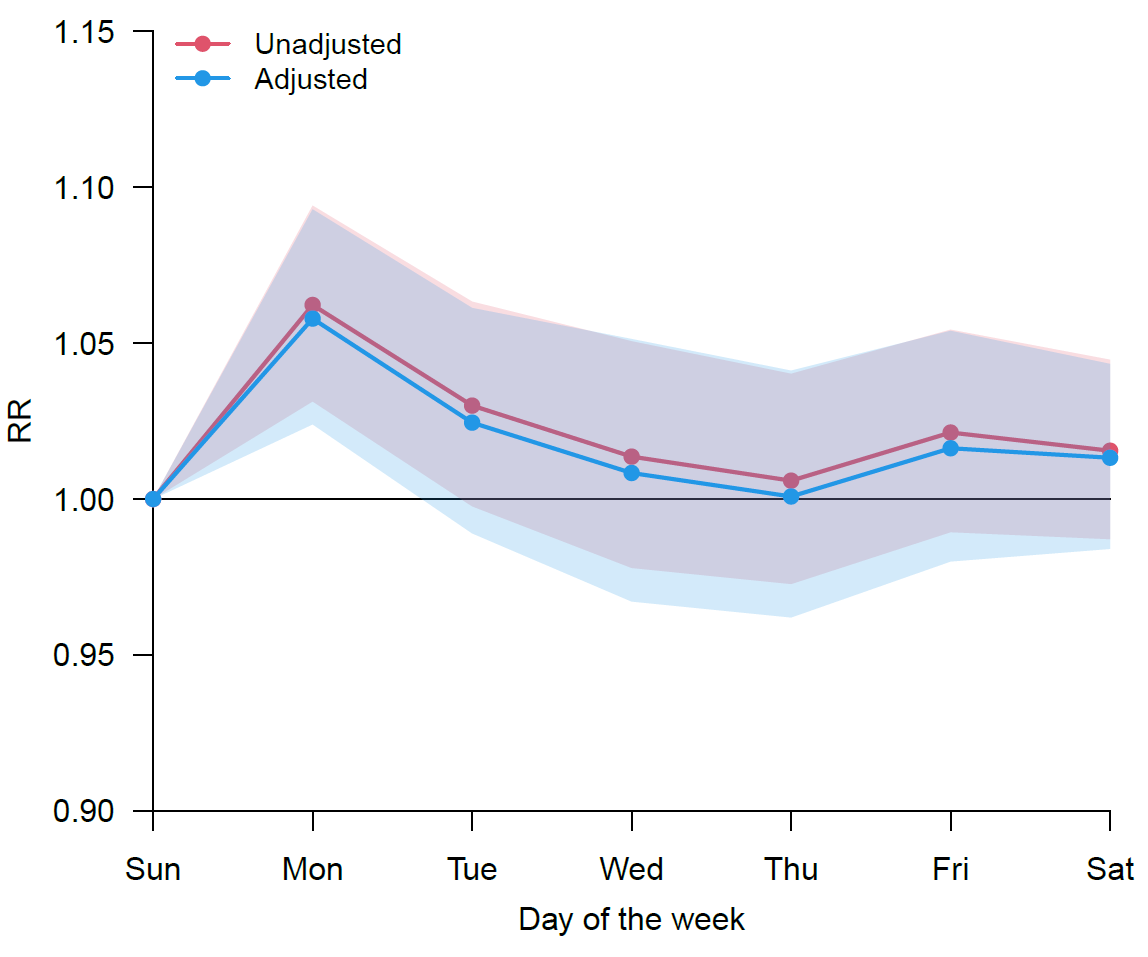 |
|  |  |  |  |
| **2. Adjusted by daily mean PM_10_ concentration** |  |  |  |
|  |  |  |  |
| a) Q1 | b) Q2 | c) Q3 | d) Q4 |
|  | |  |  |
| 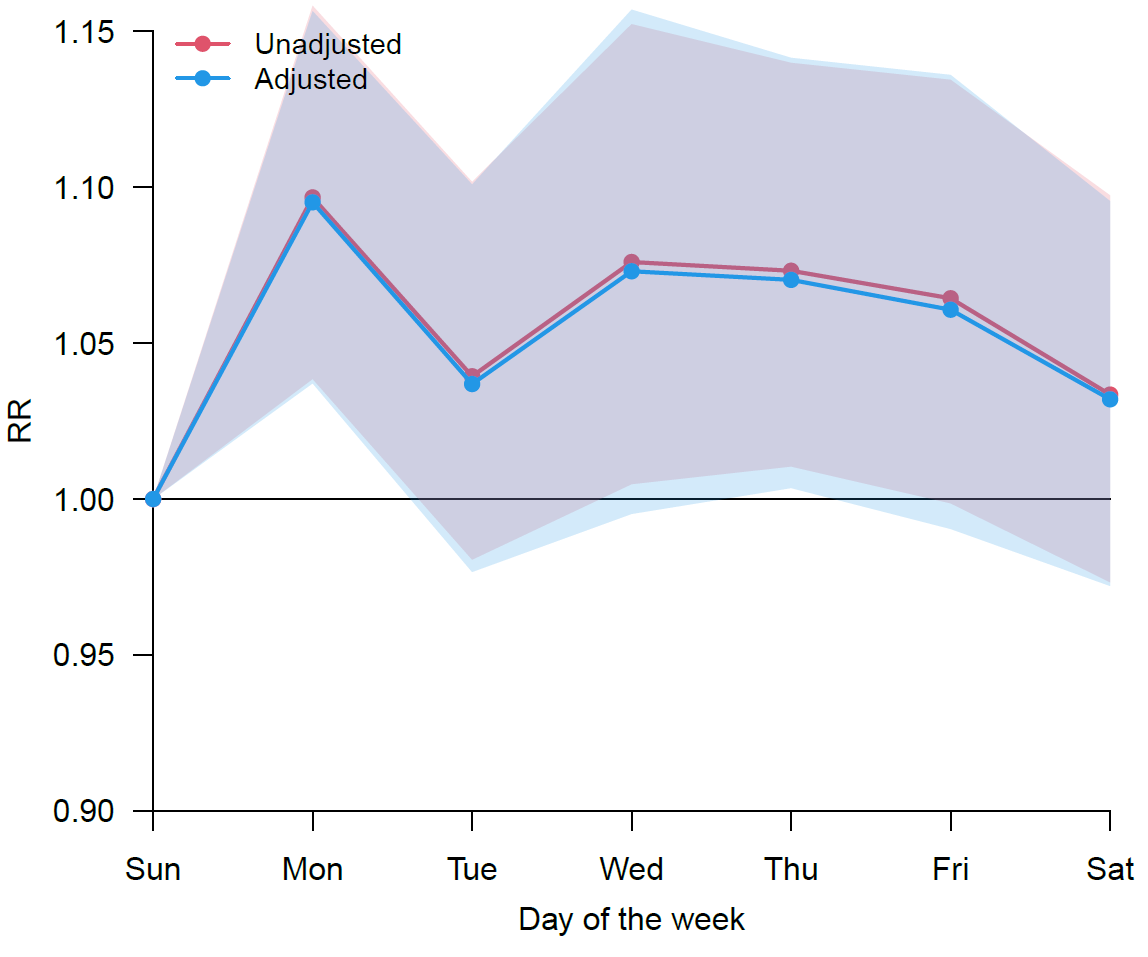 | 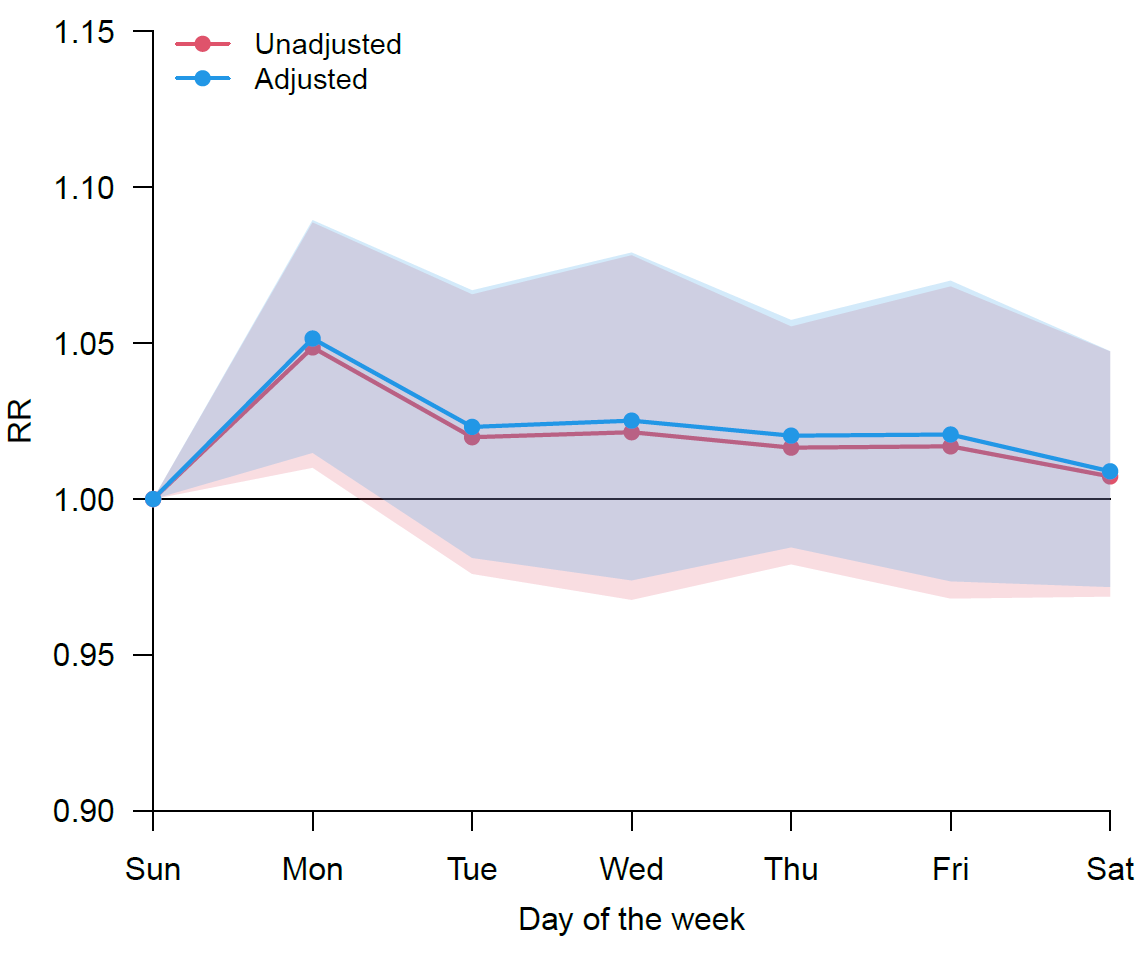 | 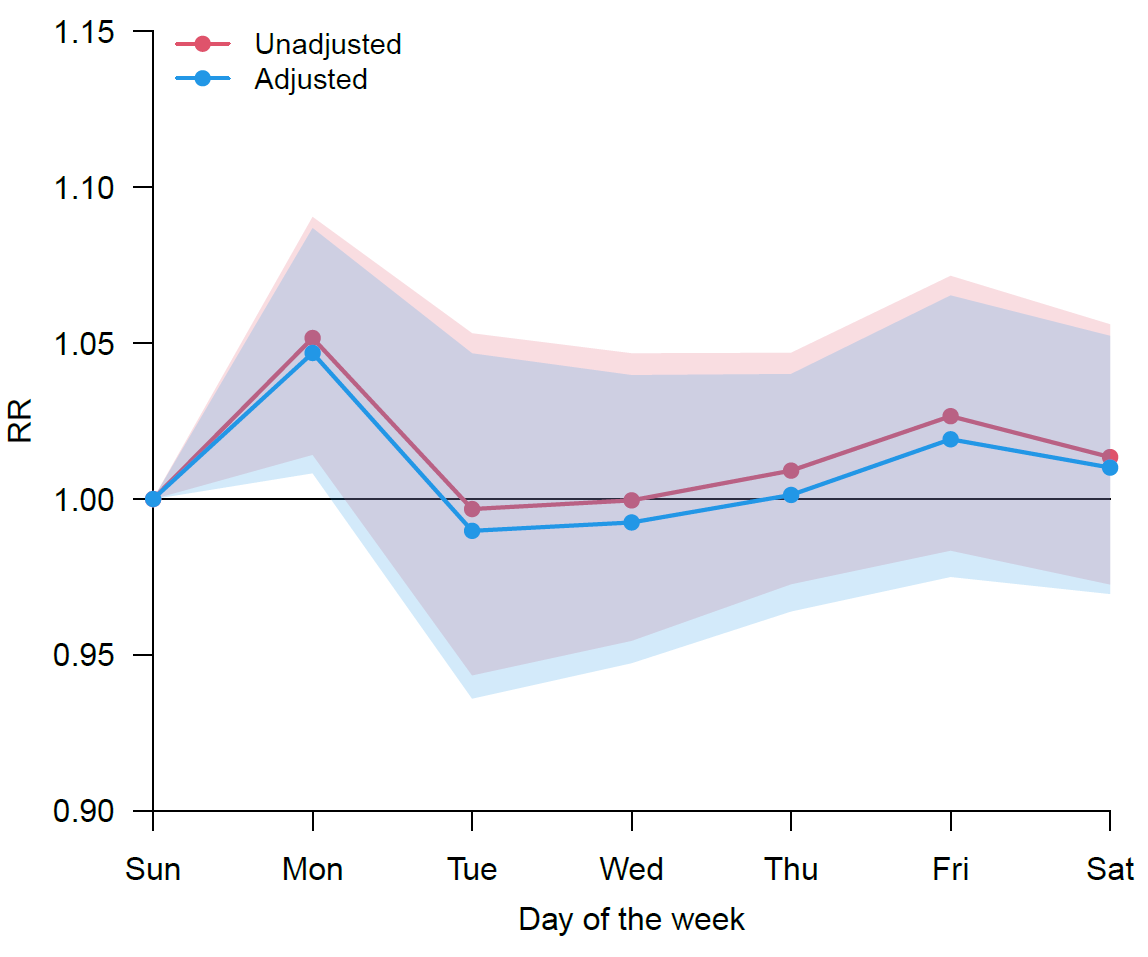 | 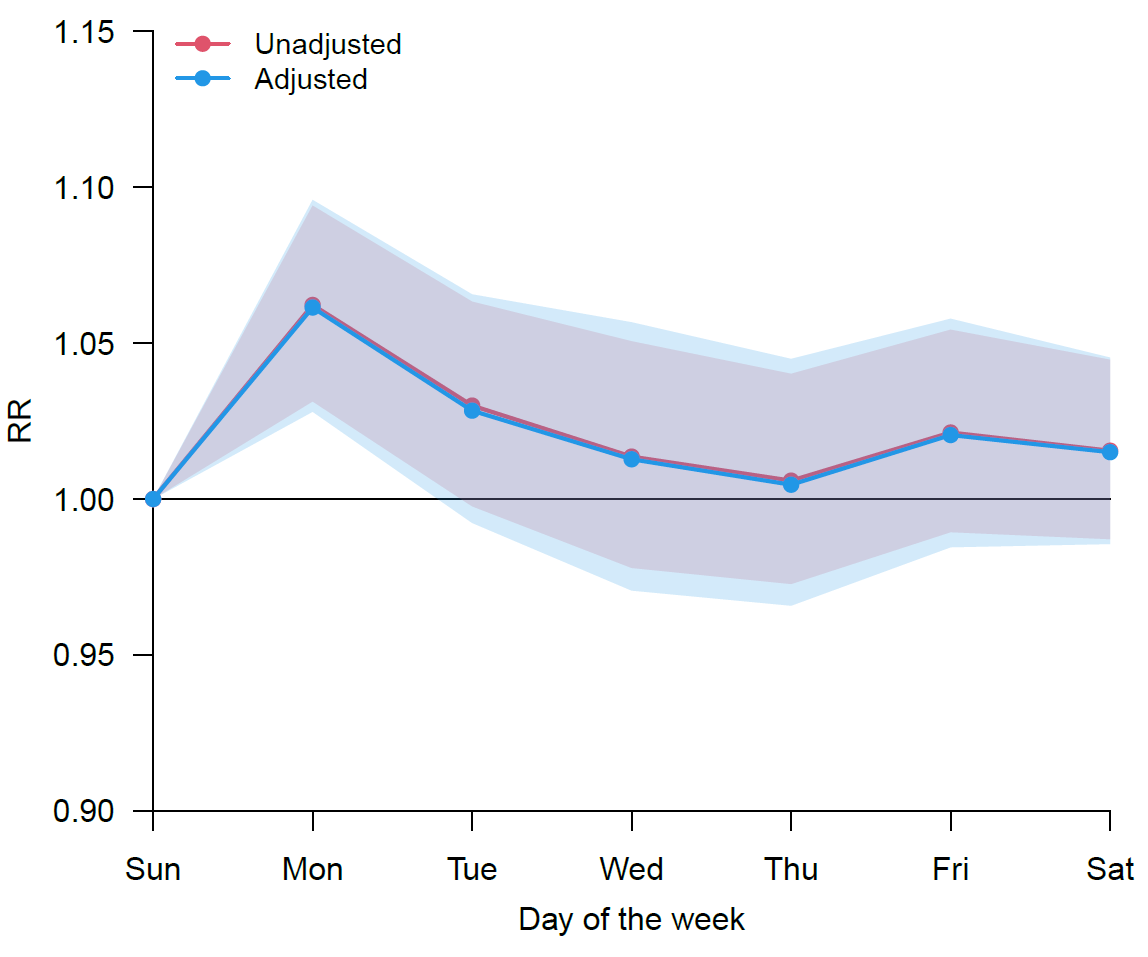 |
|  |  |  |  |
| **3. Adjusted by daily mean in NO_2_ concentration** | |  |  |
|  |  |  |  |
| a) Q1 | b) Q2 | c) Q3 | d) Q4 |
|  |  |  |  |
| 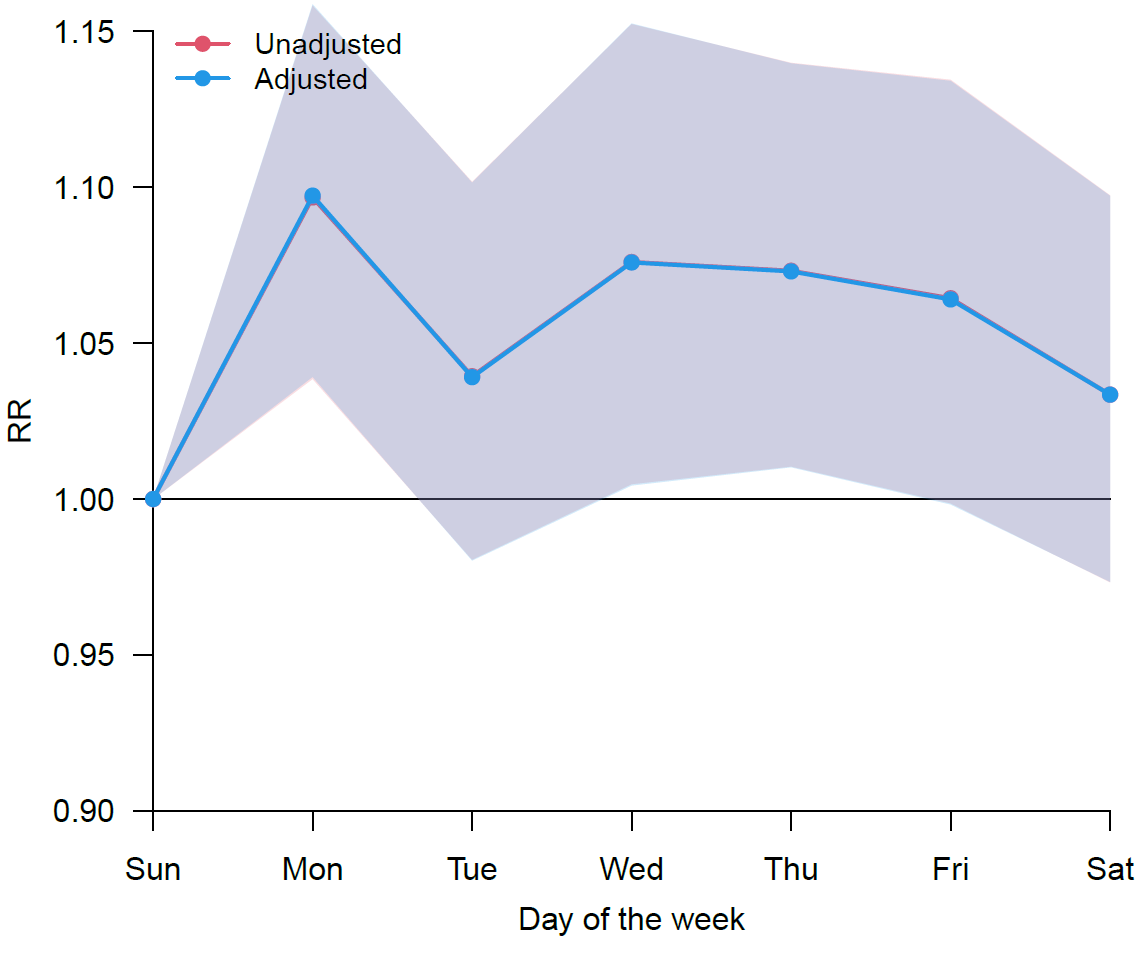 | 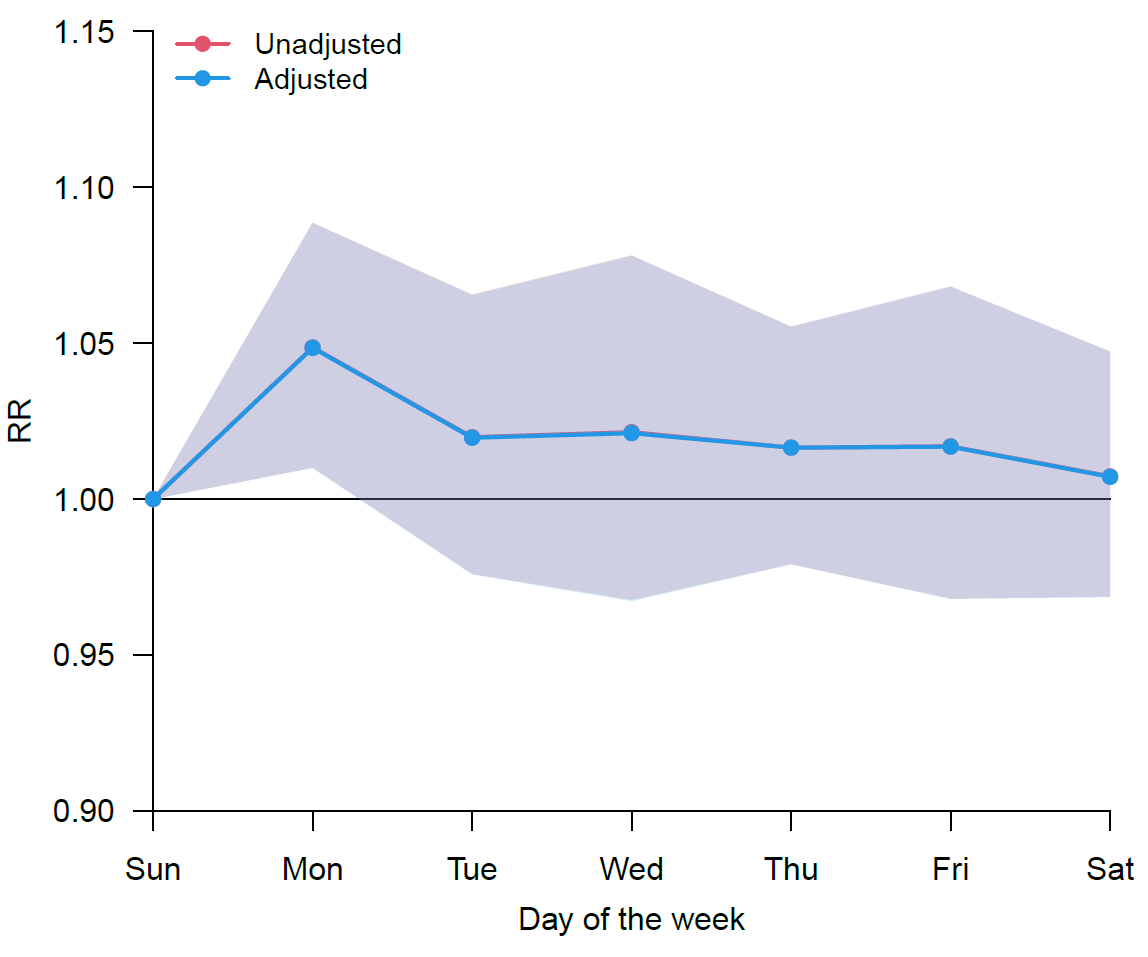 | 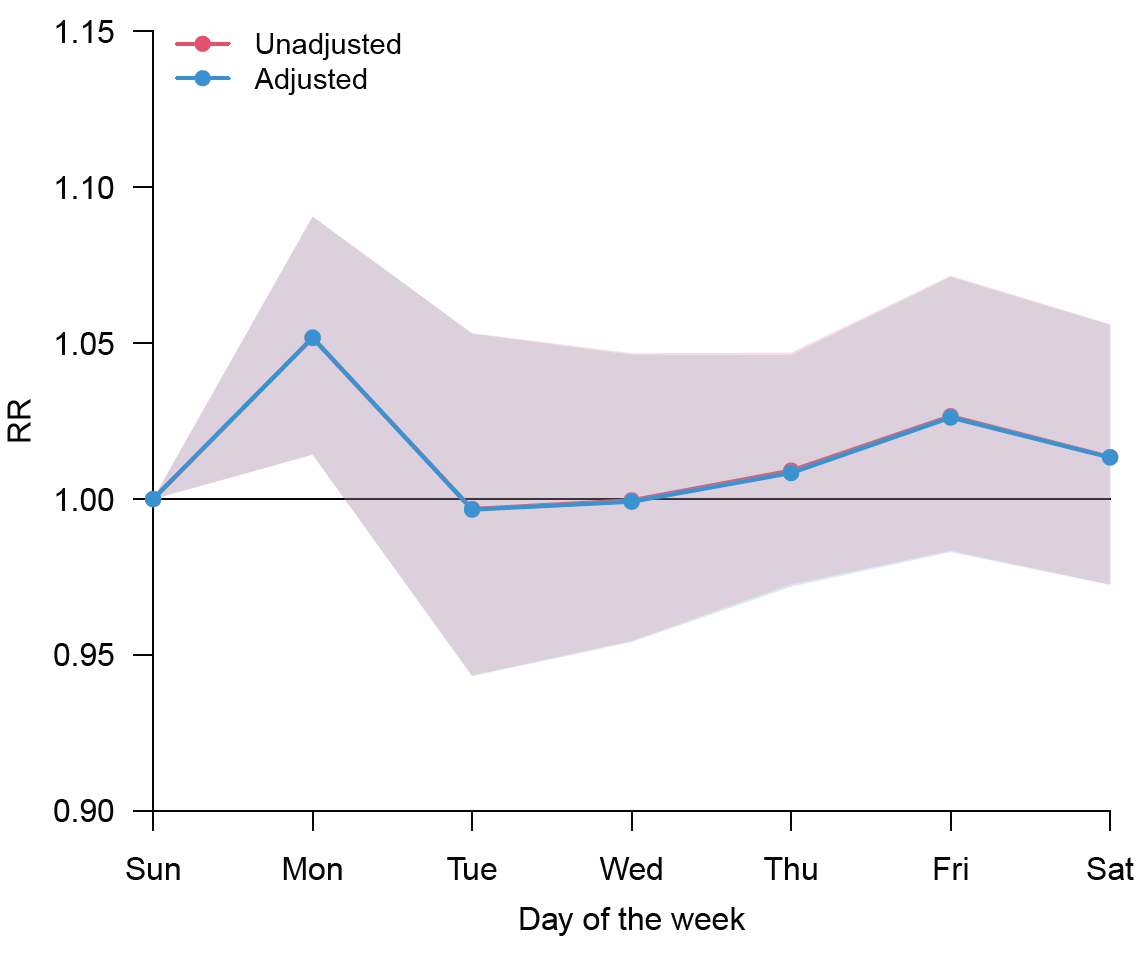 | 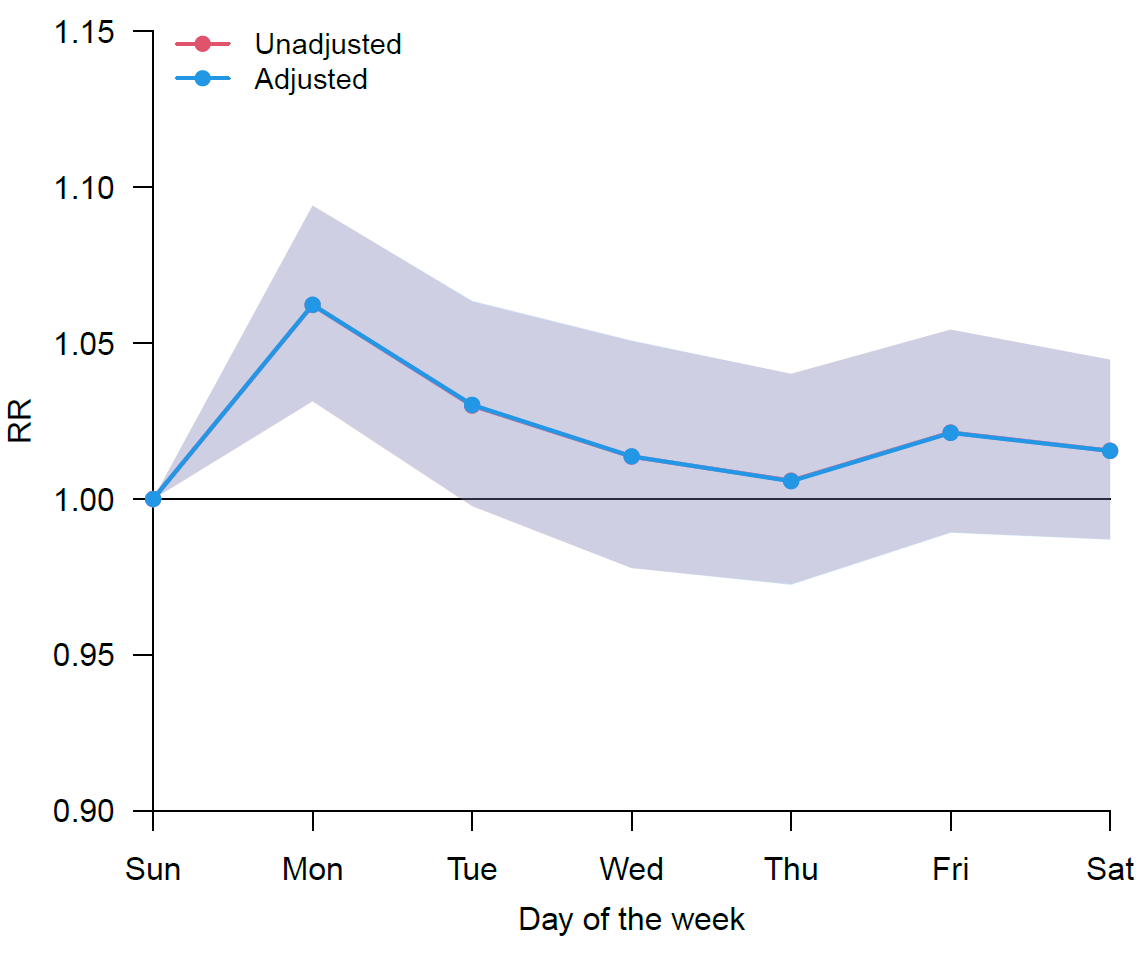 |
|  |  |  |  |
| **4. Adjusted by daily mean in O_3_ concentration** |  |  |  |
|  |  |  |  |
| a) Q1 | b) Q2 | c) Q3 | d) Q4 |
|  |  |  |  |
| 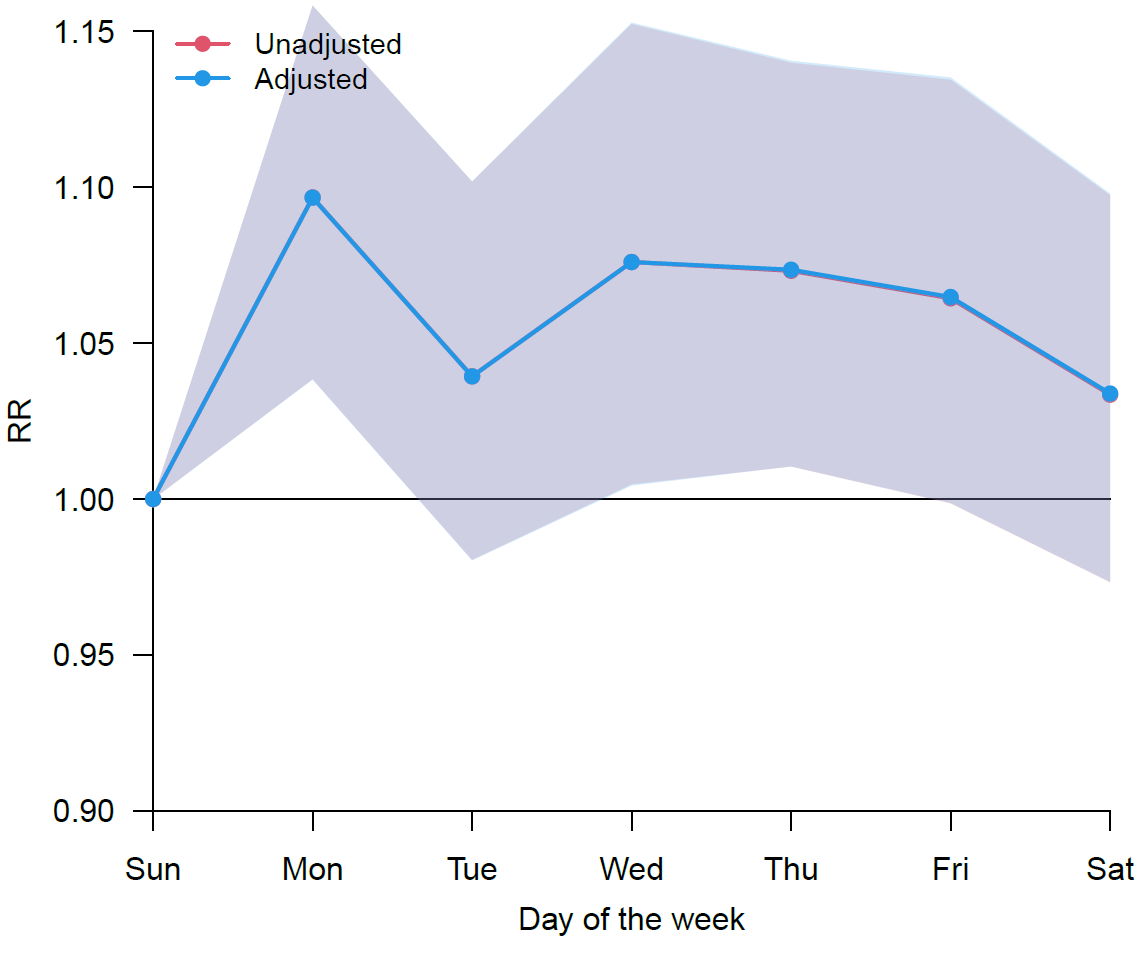 | 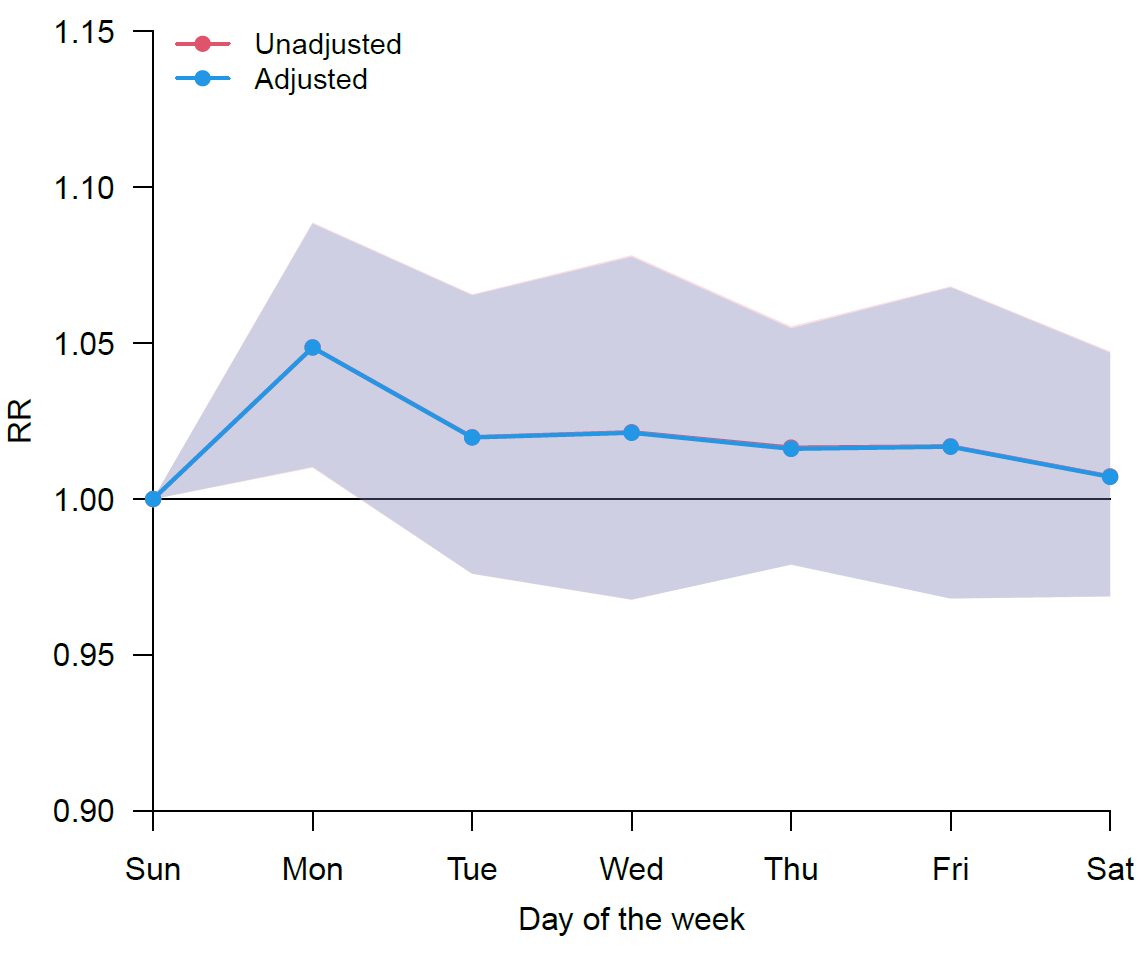 | 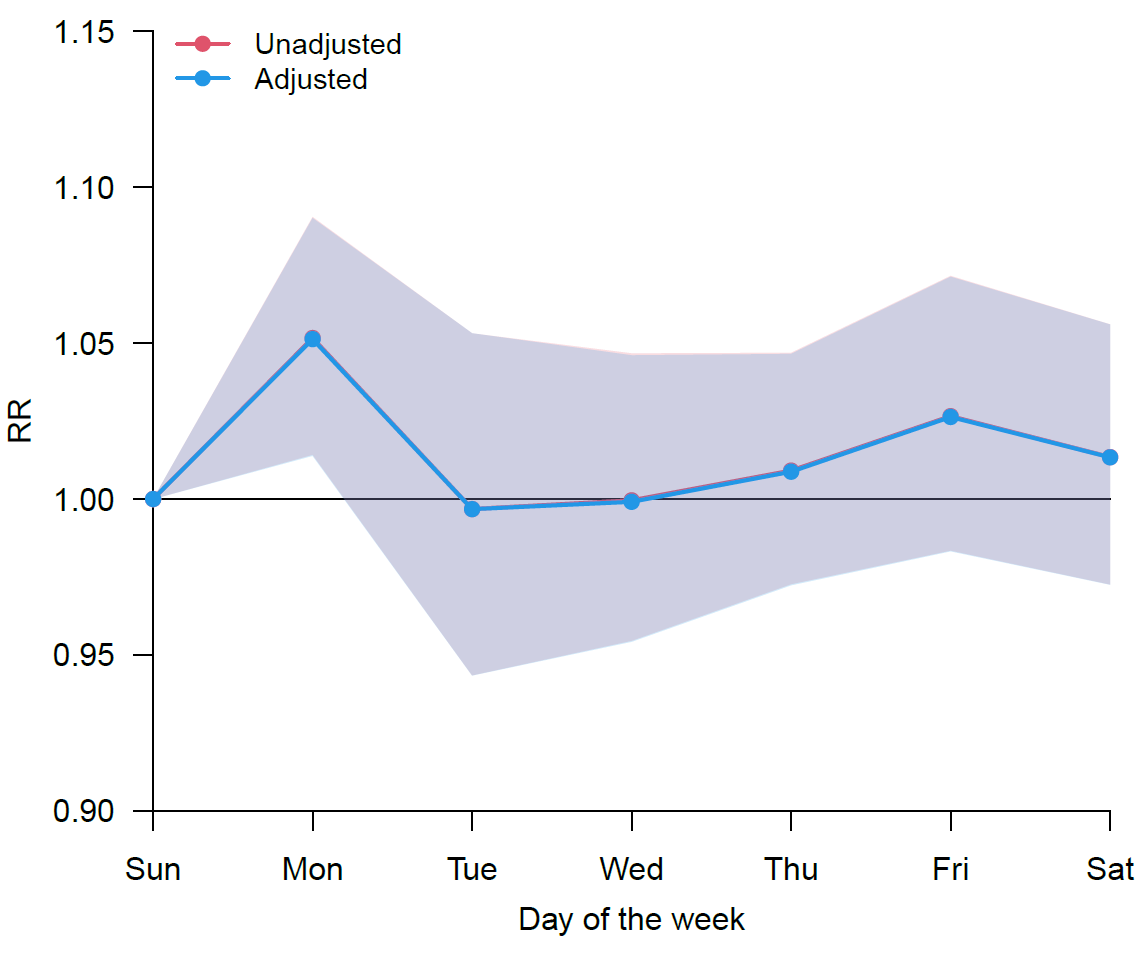 | 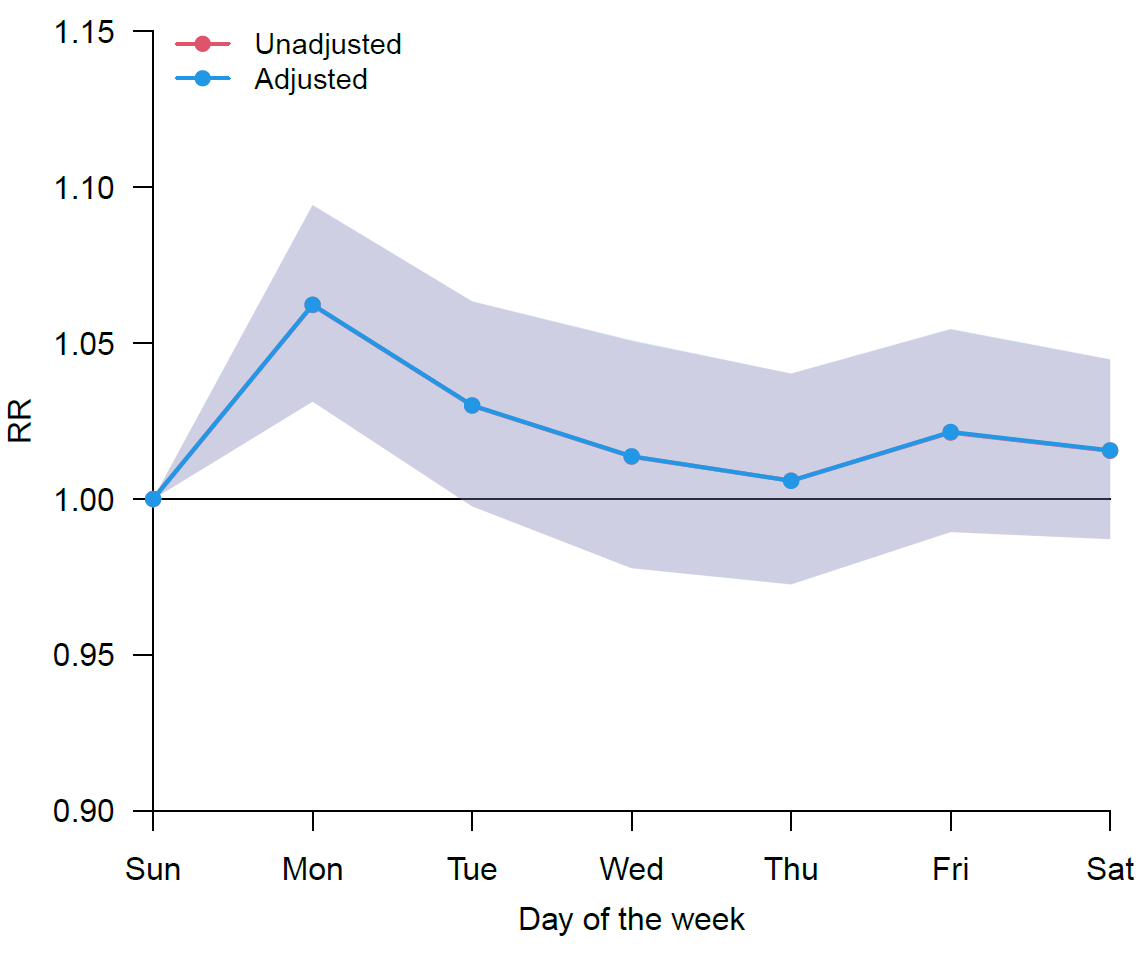 |
|  |  |  |  |

# **Supplemental Figure 8. Weekly variation in AMI mortality before and after adjustment for inter-day change in pollution by degree of urbanisation**

RR=relative risk. RR curves are computed using Sunday as a reference. Q=Quartile. Quartiles are based on population density data.

|  |  |  |  |
| --- | --- | --- | --- |
| **1. Adjusted by inter-day change in PM_2·5_ concentration** | |  |  |
|  |  |  |  |
| a) Q1 | b) Q2 | c) Q3 | d) Q4 |
|  |  |  |  |
| 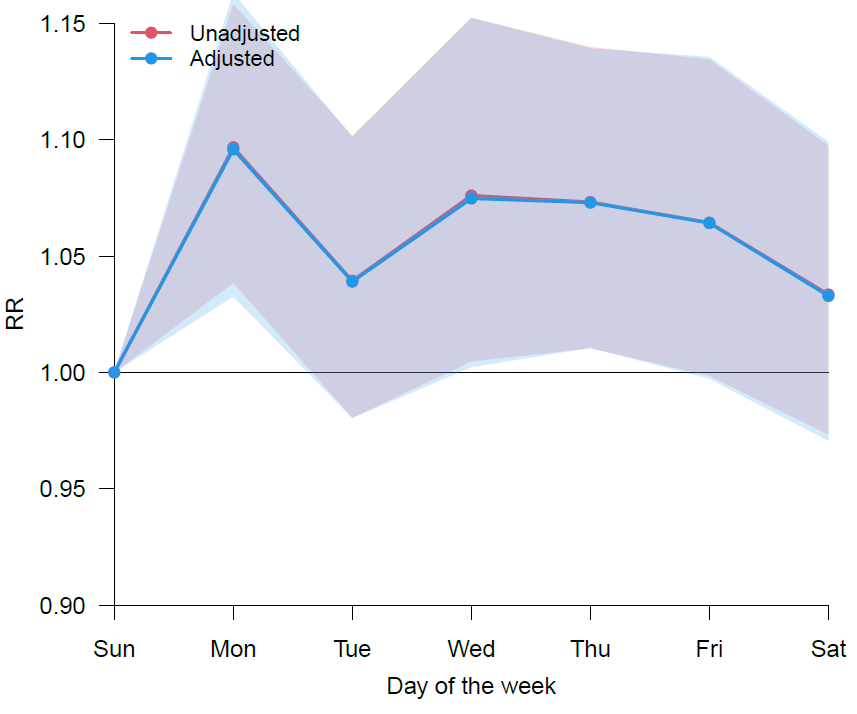 | 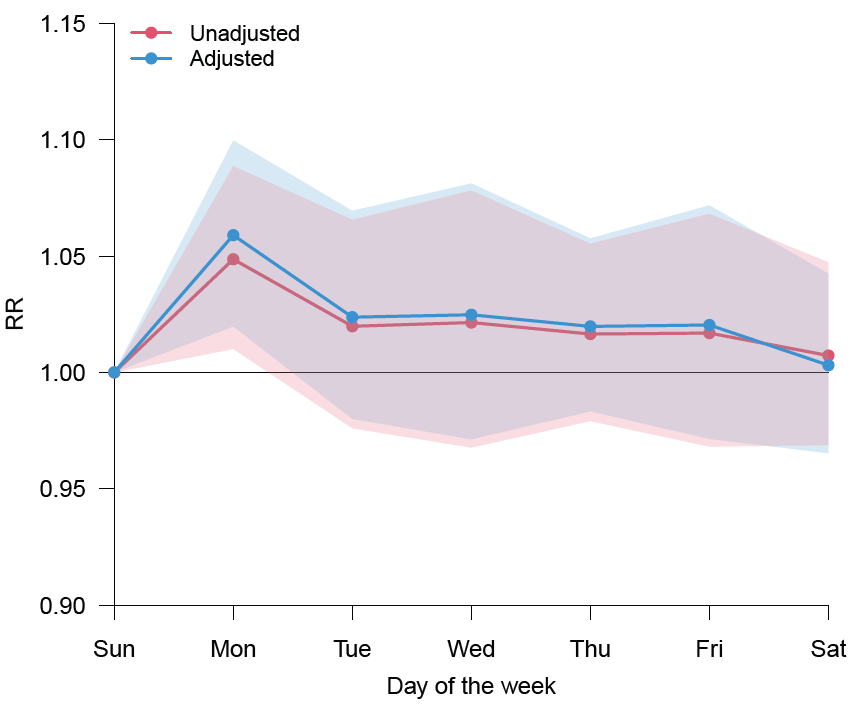 | 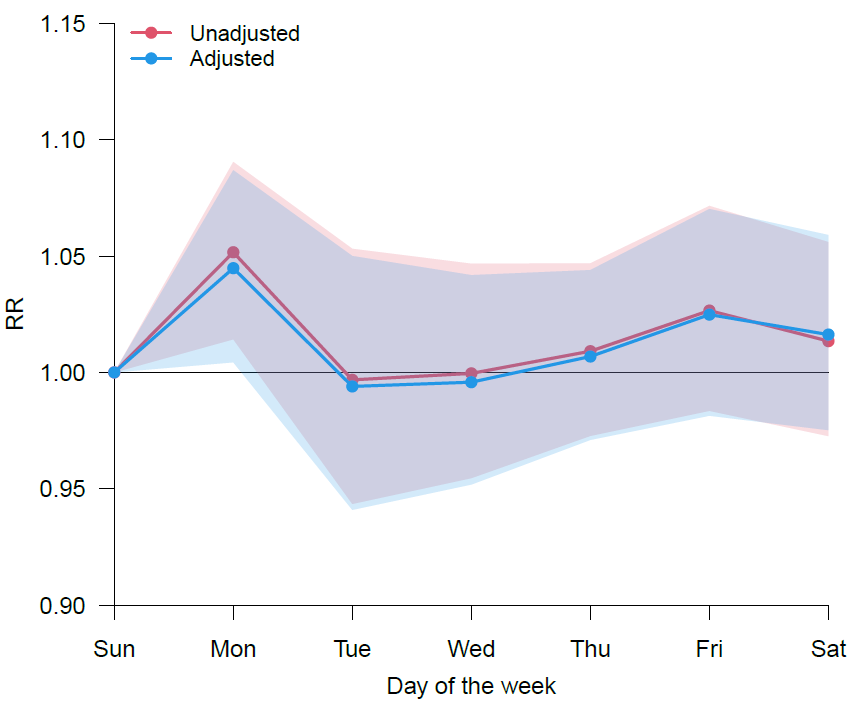 | 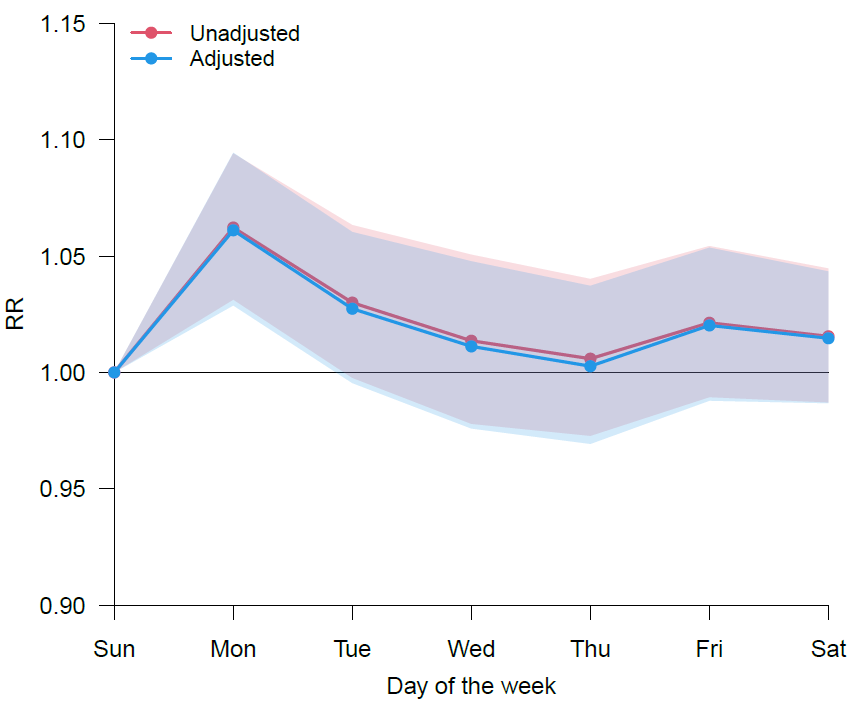 |
|  |  |  |  |
| **2. Adjusted by inter-day change in PM_10_ concentration** | |  |  |
|  |  |  |  |
| a) Q1 | b) Q2 | c) Q3 | d) Q4 |
|  | |  |  |
| 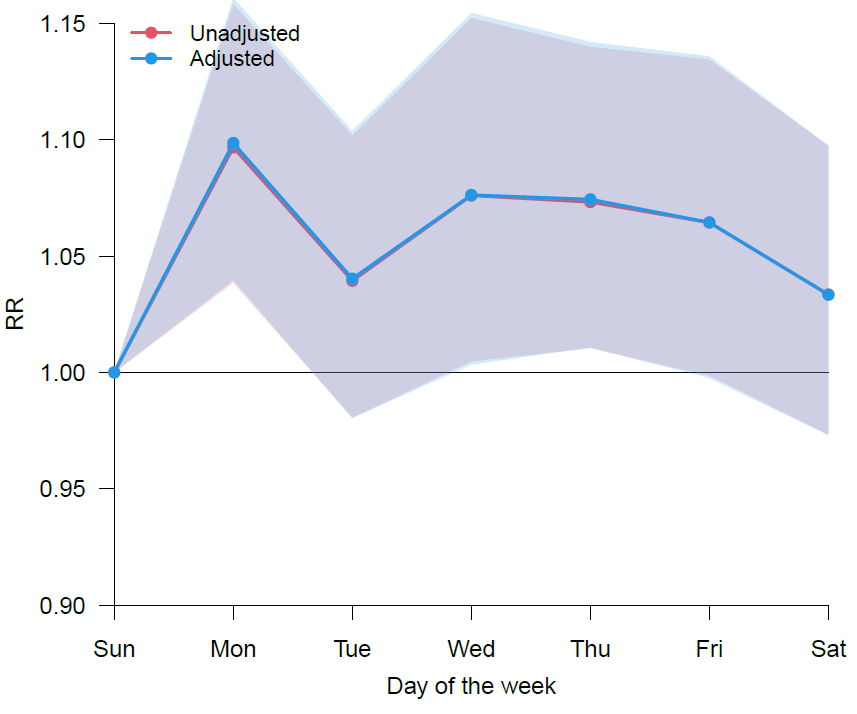 | 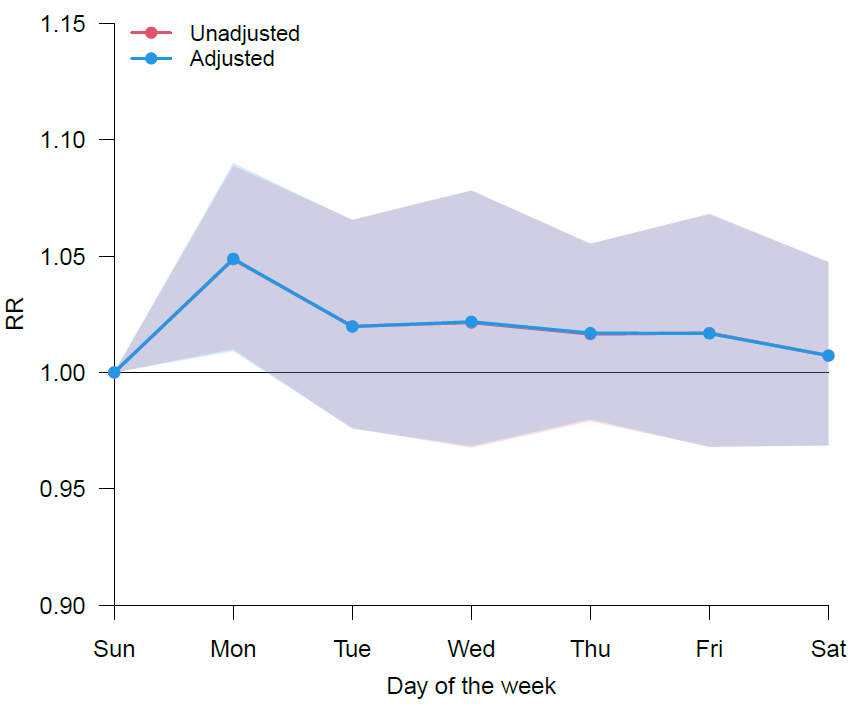 | 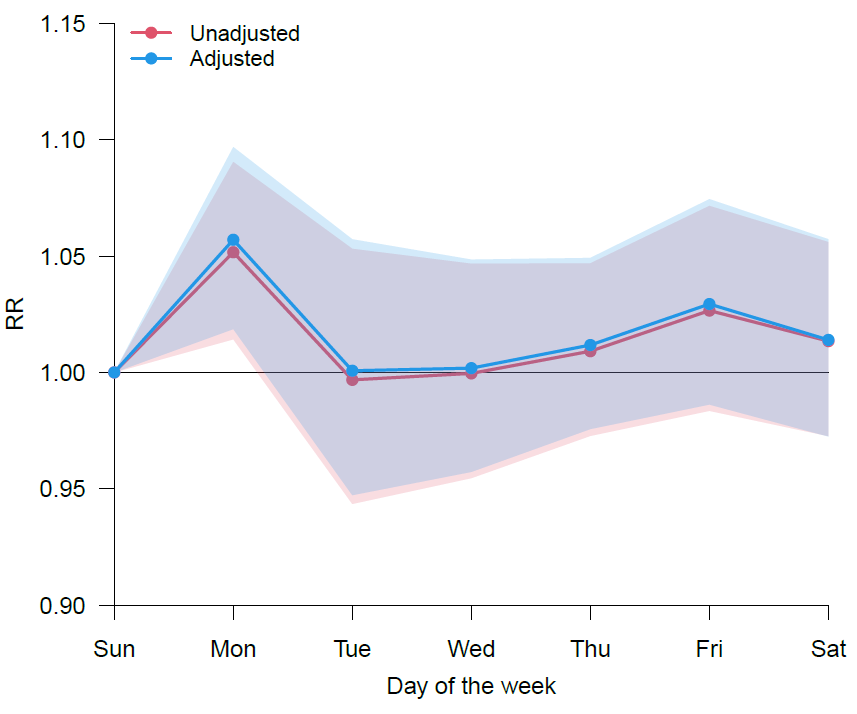 | 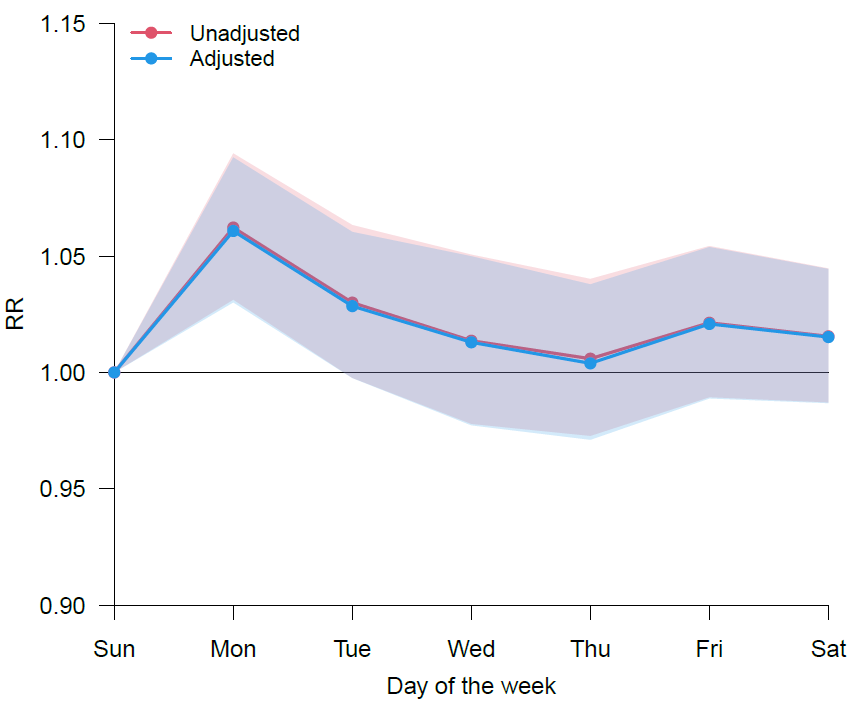 |
|  |  |  |  |
| **3. Adjusted by inter-day change in NO_2_ concentration** | |  |  |
|  |  |  |  |
| a) Q1 | b) Q2 | c) Q3 | d) Q4 |
|  |  |  |  |
| 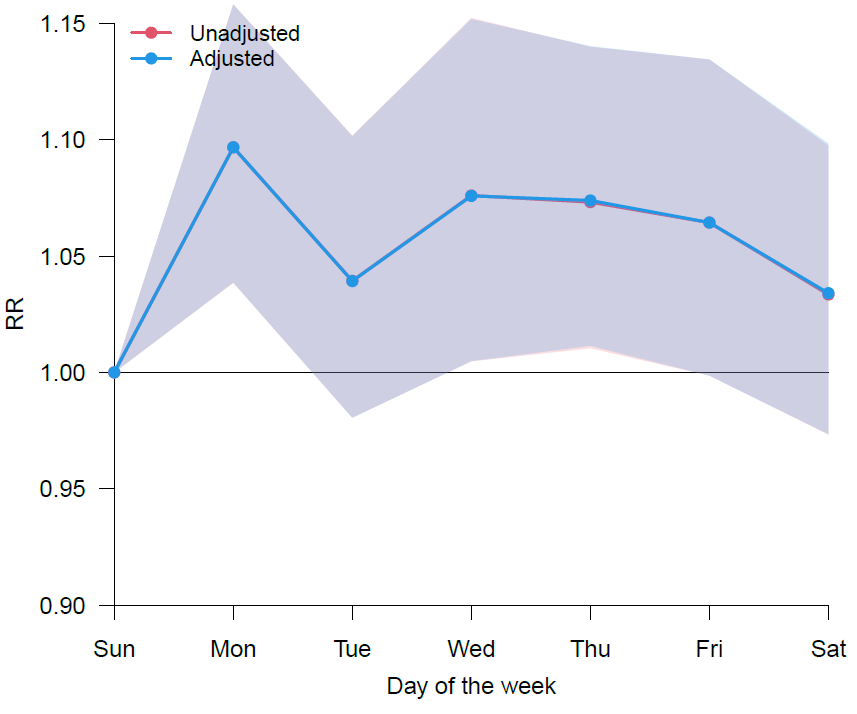 | 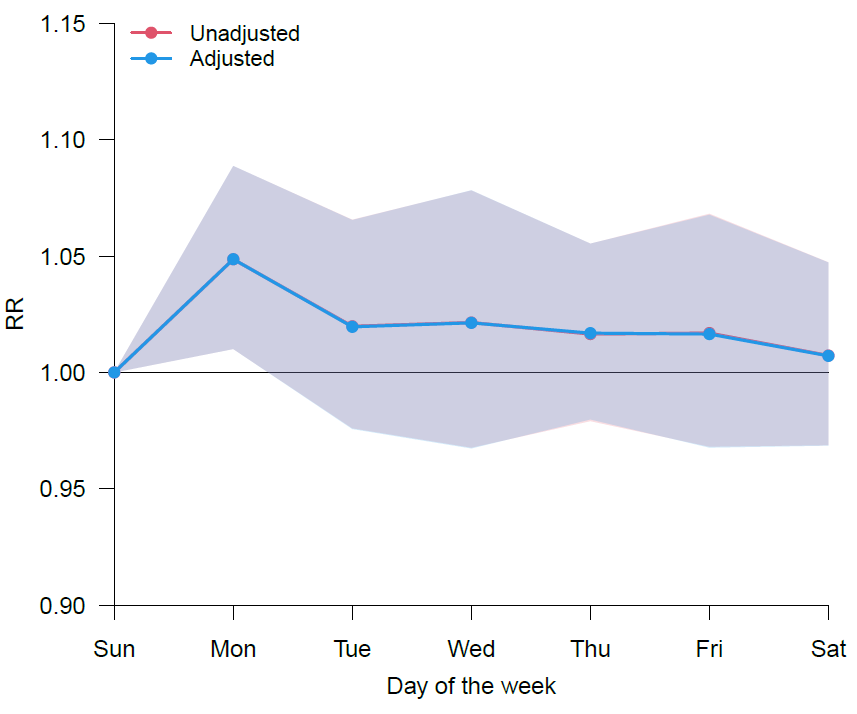 | 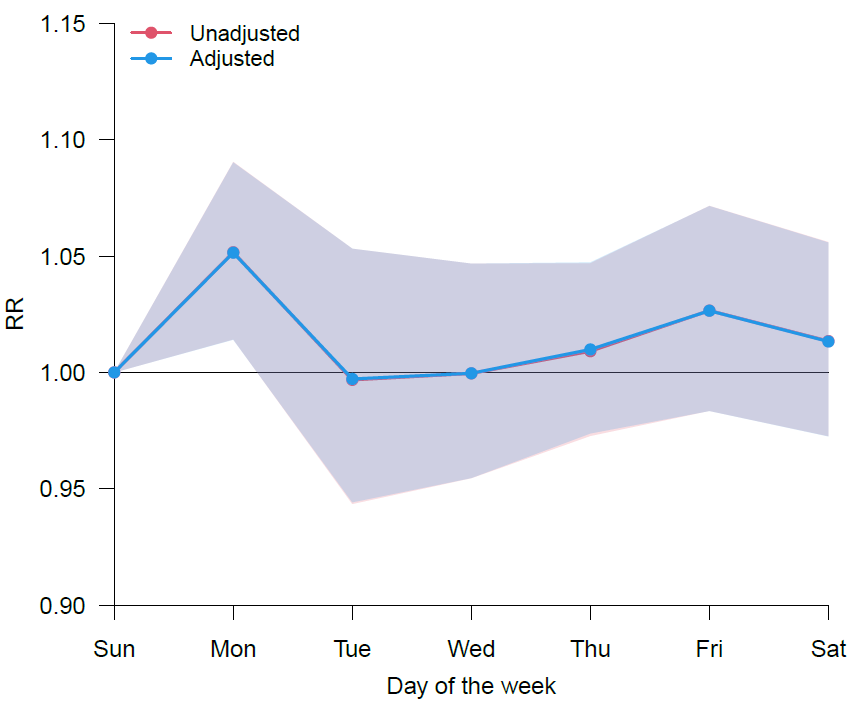 | 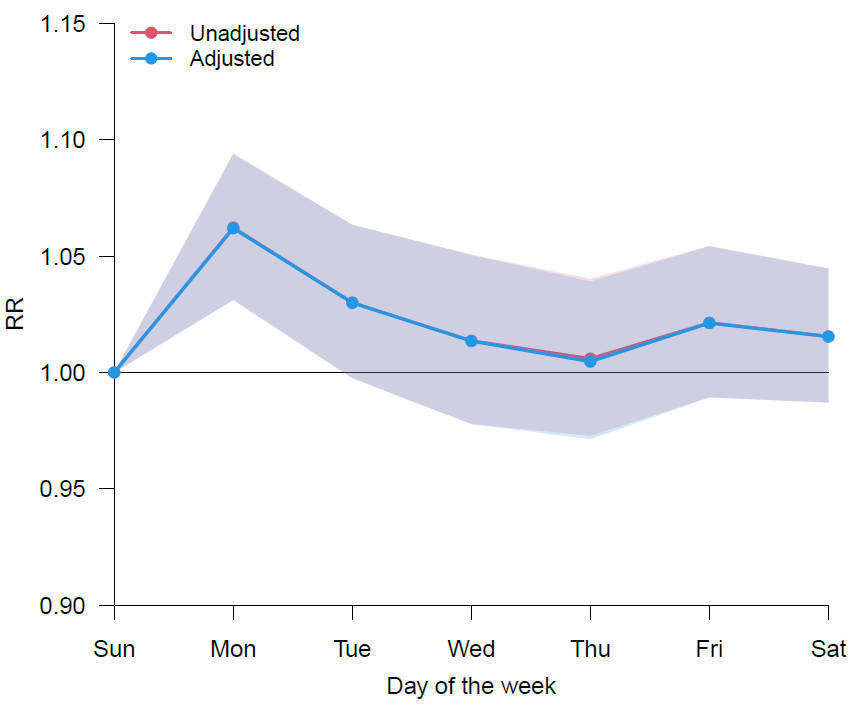 |
|  |  |  |  |
| **4. Adjusted by inter-day change in O_3_ concentration** | |  |  |
|  |  |  |  |
| a) Q1 | b) Q2 | c) Q3 | d) Q4 |
|  |  |  |  |
| 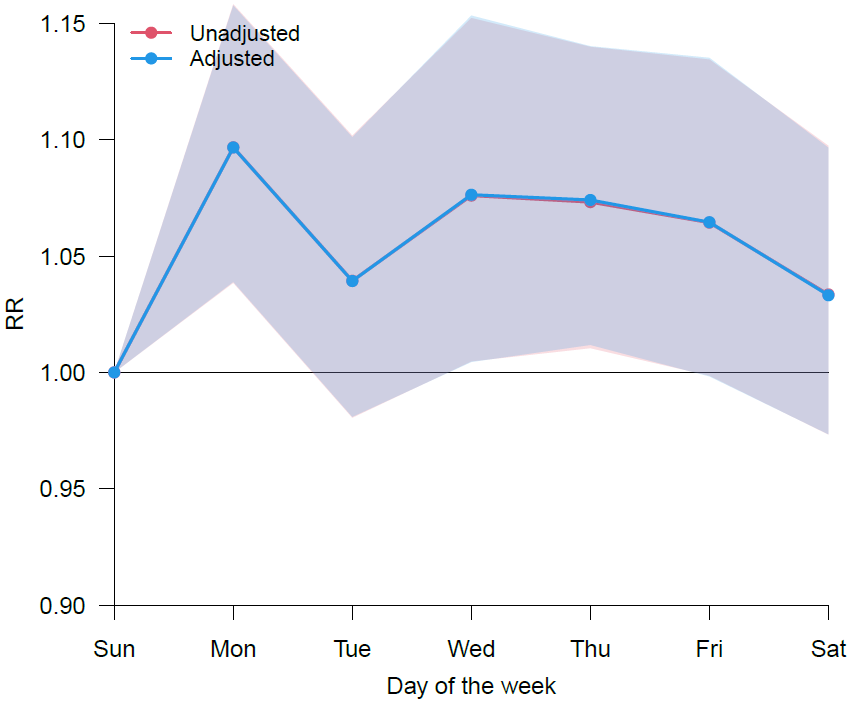 | 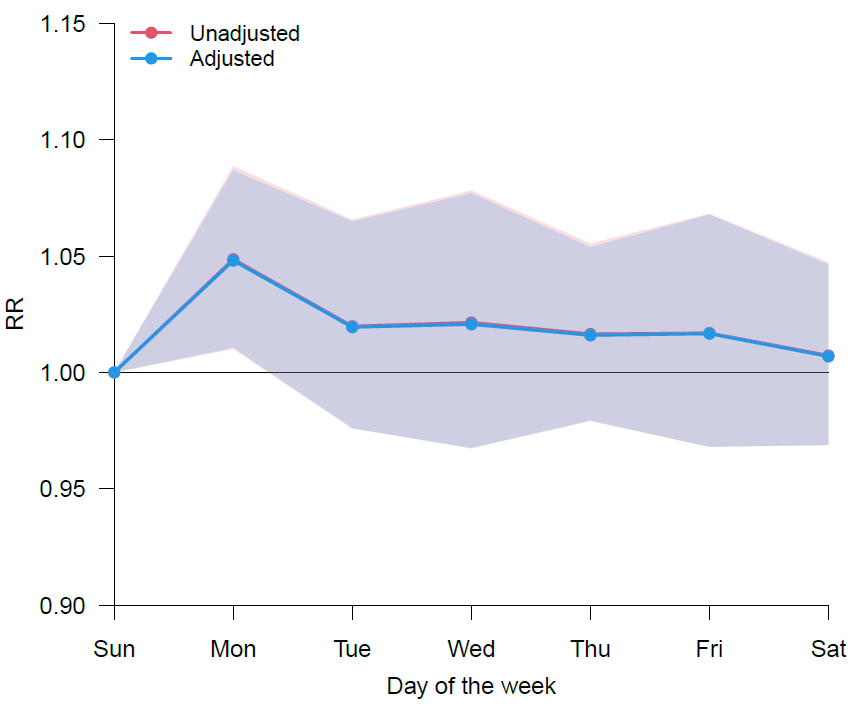 | 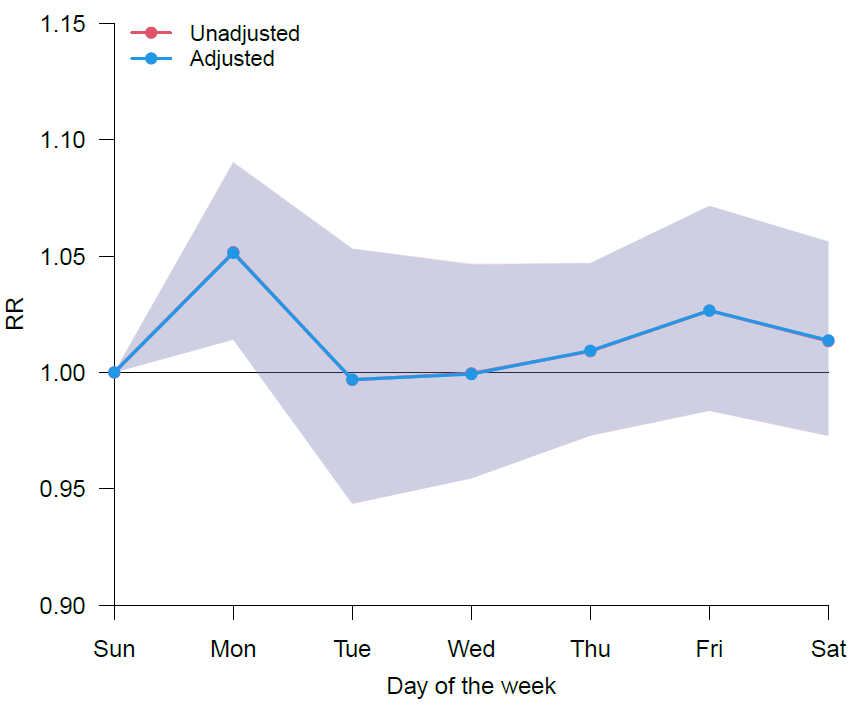 | 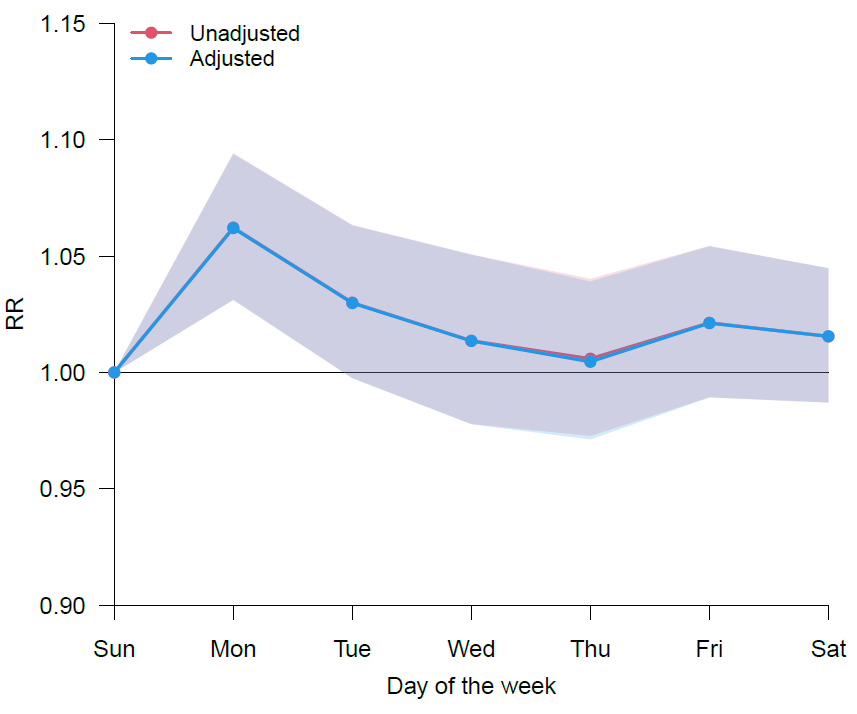 |
|  |  |  |  |

# **Supplemental Table 1. Monday excess in AMI mortality risk by degree of urbanisation**

The estimates represent the percentage increase in mortality risk per 1-μg-per-Cubic-Meter increase in air pollutants along with 95% CI. Q=Quartile. Quartiles are based on population density data.

|  |  | PM2.5 | PM10 | NO2 | O3 |
| --- | --- | --- | --- | --- | --- |
| Q1 |  | -0.03 (-5.90 to 6.21) | -0.69 (-2.88 to 1.56) | 0.55 (-1.91 to 3.08) | 0.28 (-0.85 to 1.43) |
| Q2 |  | 0.00 (-1.58 to 1.60) | 0.26 (-0.23 to 0.76) | 0.84 (-0.07 to 1.76) | -0.19 (-0.49 to 0.12) |
| Q3 |  | -2.08 (-3.79 to -0.34) | -0.22 (-2.29 to 1.90) | 1.67 (0.22 to 3.14) | 0.14 (-0.51 to 0.79) |
| Q4 |  | 0.29 (-1.23 to 1.84) | 0.41 (-0.19 to 1.01) | 0.74 (-0.19 to 1.68) | -0.09 (-0.68 to 0.49) |

# **Supplemental Table 2. Analysis of heterogeneity from multivariate meta-analysis of the weekly variation in AMI mortality**

Cochran Q test for heterogeneity, I^2^ statistics for residual heterogeneity.

|  |  | Daily AP | |  | Inter-day change in AP | |
| --- | --- | --- | --- | --- | --- | --- |
|  |  | Q test | I2 |  | Q test | I2 |
| Model |  | (p-value) | (%) |  | (p-value) | (%) |
| Unadjusted |  | 0.156 | 7.8 |  | 0.156 | 7.8 |
| PM2.5 adjusted |  | 0.074 | 11.1 |  | 0.160 | 7.7 |
| PM10 adjusted |  | 0.08 | 10.7 |  | 0.206 | 6.4 |
| NO2 adjusted |  | 0.154 | 7.9 |  | 0.153 | 7.9 |
| O3 adjusted |  | 0.155 | 7.9 |  | 0.153 | 7.9 |

# **Supplemental Table 3. Analysis of heterogeneity from meta-analysis the association between air pollution and Monday peak in AMI**

Cochran Q test for heterogeneity, I^2^ statistics for residual heterogeneity.

|  |  | Q test | I2 |
| --- | --- | --- | --- |
| Model |  | (p-value) | (%) |
| PM2.5 |  | 0.005 | 37.9 |
| PM10 |  | 0.001 | 44.2 |
| NO2 |  | 0.004 | 38.8 |
| O3 |  | 0.005 | 38.0 |
